# Gecko‐Inspired Adhesive for Robotic Grippers with Excellent Ultra‐Low‐Temperature Adhesion Performance

**DOI:** 10.1002/advs.202515084

**Published:** 2025-11-14

**Authors:** Jiabao Feng, Hong Zhu, Yadong Lv, Qi Yang, Guangxian Li, Miqiu Kong, Wei Pu

**Affiliations:** ^1^ School of Aeronautics and Astronautics Key Laboratory of Advanced Spatial Mechanism and Intelligent Spacecraft Ministry of Education National Key Laboratory of Advanced Polymer Materials Sichuan University Chengdu 610065 P. R. China; ^2^ College of Polymer Science and Engineering National Key Laboratory of Advanced Polymer Materials Sichuan University Chengdu 610065 P. R. China

**Keywords:** adhesion mechanism, bioinspired adhesion, microstructure, ultra‐low temperature

## Abstract

Gecko‐inspired adhesives, which are traditionally based on polydimethylsiloxane (PDMS), are suitable for the delicate handling of 3D objects under vacuum and microgravity conditions but lose almost all their adhesion strength in environments below their crystallization temperatures. To address this issue, poly(methyl‐phenyl‐vinyl)siloxane (PMPVS) is synthesized by the simultaneous introduction of phenyl and vinyl side groups to achieve a crystallization temperature of −93.0 °C. The developed PMPVS‐based adhesive exhibited adhesion strengths of 25.9 and 36.7 kPa at −70 and −80 °C, respectively, while a conventional PDMS‐based adhesive has an adhesion strength of only 0.7 kPa at −70 °C. Moreover, the PMPVS‐based adhesive retained an adhesion strength of 29.4 kPa at −80 °C even after 100 cycles. Robotic grippers equipped with this adhesive can softly grasp irregular, fragile, and heavy objects with a reduced gripping force of up to 90% compared to grippers without adhesive over a wide temperature range.

## Graphical abstract

Phenyl and vinyl groups are used to modify conventional polydimethylsiloxane (PDMS) to yield poly(methylphenylvinylsiloxane) (PMPVS). PMPVS‐based gecko‐inspired arrays with mushroom tips exhibit superior adhesion strength of 36.7 kPa even at −80 °C, which enhances by 5143% compared to PDMS‐based arrays (0.7 kPa at −70 °C). This work significantly broadens the applications of gecko‐inspired adhesives into an extremely low temperature range.

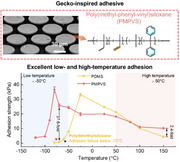

## 1 Introduction

The development of gecko‐inspired adhesives has facilitated the design of advanced robotic grippers capable of performing delicate and complex tasks.^[^ ^1^, ^2^, ^3^ ^]^ Gecko‐inspired adhesives have emerged as a promising alternative to vacuum,^[^ ^4^ ^]^ chemical,^[^ ^5^ ^]^ magnetic,^[^ ^6^ ^]^ and electrostatic^[^ ^7^ ^]^ methods that can address long‐standing technical bottlenecks in compliant grasping, 3D manipulation, the handling of heavy payloads, and miniature robotic systems. Gecko‐inspired adhesives work by exploiting van der Waals forces and offer multiple advantages: universality (i.e., capability of adhering to diverse surfaces including noncooperative targets), reversible and repeatable adhesion, nondestructive contact for low‐impact docking, robust environmental adaptability, passive nature (i.e., no external energy source required), and simple design.^[^ ^8^, ^9^ ^]^ However, the integration of gecko‐inspired adhesives into robotic grippers remains constrained by their insufficient adhesion strength, limited improvement of the gripping force, and low adaptability to 3D objects, especially at low environmental temperatures.

To overcome these challenges, three key aspects have been explored^[^ ^10^ ^]^: a uniform normal stress distribution, shear load sharing, and the contact area. The adhesion strength of gecko‐inspired adhesives decreases with increasing area scale because of an uneven load distribution. To achieve a near‐uniform load distribution and improve adaptability, gecko‐inspired adhesives are usually segmented into independent zones supported by hyper‐elastic components,^[^ ^11^ ^]^ cast in Kapton films,^[^ ^12^ ^]^ embedded in polydimethylsiloxane (PDMS) films,^[^ ^13^ ^]^ or integrated with electrostatic interactions.^[^ ^14^ ^]^ Furthermore, shear load sharing is necessary to adapt to high‐curvature or irregular objects and to withstand lateral loads.^[^ ^10^, ^15^ ^]^ Moreover, increasing the contact area between a gecko‐inspired adhesive and the target object through structural optimization can promote shear load sharing and an even stress distribution, and thus facilitate the stable and gentle grasping of heavy, large, and fragile 3D objects even in microgravity.

However, conventional gecko‐inspired adhesives based on PDMS lose adhesion strength at temperatures lower than about −50 °C (0.23 kPa at −80 °C),^[^ ^16^ ^]^ which can be attributed to a loss of flexibility and resilience below their crystallization temperatures.^[^ ^17^, ^18^, ^19^ ^]^ This severely limits their utility in extremely low‐temperature environments such as polar regions and space. To lower the crystallization temperature, Xia et al.^[^ ^16^ ^]^ introduced phenyl groups into the molecular structure of poly(methyl‐phenyl)siloxane (PMPS)‐based gecko‐inspired adhesives, which enhances the adhesion strength at −120 °C (1.06 kPa) by 30.9% compared with the adhesion strength at 25 °C (0.81 kPa). However, the reduced compliance of PMPS induced by the increased molecular rigidity by introduced phenyl groups meant that the adhesion strength is still relatively low at −120 °C and insufficient for practical use. Zhang et al.^[^ ^20^ ^]^ modified PDMS with electrically conductive carbon black, which can be electric heat to ensure the elasticity of the adhesive at low temperatures. However, this process is complex and time‐consuming due to the electrical stimulation using additional equipment. Thus, the application of gecko‐inspired adhesives in low‐temperature environments is still a major challenge.

In this work, we synthesized poly(methyl‐phenyl‐vinyl)siloxane (PMPVS) by the simultaneous introduction of phenyl and vinyl side groups and developed a gecko‐inspired adhesive with high adhesion strength at low temperatures. Experiments, molecular dynamics simulation, and finite element analysis were conducted to evaluate the adhesion performance of the PMPVS‐based adhesive at low temperatures compared with the commercial PDMS‐based adhesive and evaluate its potential for practical application in low‐temperature environments.

## 2 Results and Discussion

### 2.1 Design and Characterization

**Figure 1:**
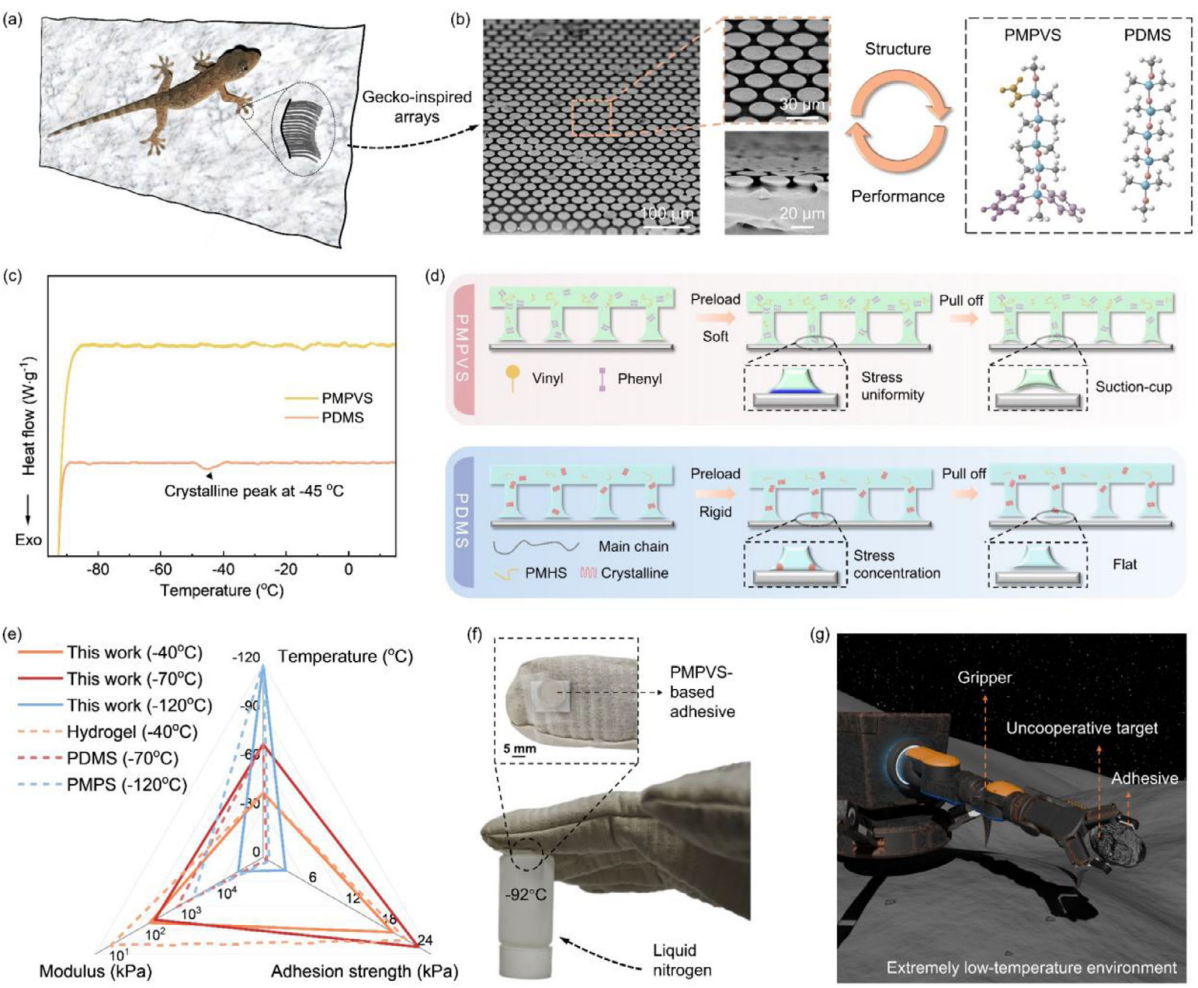
Design of gecko‐inspired adhesives for low‐temperature environments. a) Schematic of a gecko climbing vertically on the wall and the micro‐ and nano‐structures of the gecko's foot. b) Morphology of the gecko‐inspired adhesive and molecular structures of PMPVS and PDMS. c) The crystallinity curves of PMPVS and PDMS from −95 to 20 °C. d) Changes in PMPVS‐ and PDMS‐ based adhesives during adhesion at extremely low temperatures. e) Comparison of adhesion strength and modulus of the PMPVS‐based adhesive at −40 °C (Orange solid line), −70 °C (Red solid line), and −120 °C (Blue solid line) with these of hydrogel at −40 °C (Orange dashed line),^[^ ^16^ ^]^ PDMS at −70 °C (Red dashed line), and PMPS at −120 °C (Blue dashed line),^[^ ^27^ ^]^ respectively. f) Schematic of the PMPVS‐based adhesive gripping a glass bottle filled with liquid nitrogen (20 g). g) Potential applications of the PMPVS‐based adhesive in low‐temperature environments.

**Figure** **1** shows the design and characteristics of the PMPVS‐based gecko‐inspired adhesive. Geckos can adhere to vertical surfaces and climb at a speed of over 1 m s^−1^ owing to millions of setae (3–130 µm in length) on their feet with even smaller spatula (0.2–0.5 µm in diameter) at their ends (Figure 1a).^[^ ^21^ ^]^ Gecko‐inspired adhesives mimic these micro‐ and nanostructures by utilizing mushroom‐shaped arrays comprising vertical pillars with wide and flat tips that facilitate an even stress distribution^[^ ^22^, ^23^ ^]^ and increased contact area with the target surface owing to the adaptable shape of the tips^[^ ^24^ ^]^ (Figure 1b). In this study, the mushroom‐shaped arrays of the PMPVS‐based gecko‐inspired adhesive were fabricated by double‐sided photolithography,^[^ ^25^ ^]^ development, mold gating, and demolding (Figure S4, Supporting Information). The vertical pillars had a height and diameter of 4.5 and 33 µm, respectively, and the tips had a diameter of 44 µm. For gecko‐inspired adhesives based on common PDMS, the crystallization temperature (*T_c_*) was ≈−45 °C (Figure 1c), below which the resilience lost due to increased modulus. This resulted in the failure of the adhesion performance of traditional PDMS‐based gecko‐inspired adhesives at temperatures below *T_c_*.^[^ ^16^ ^]^ To address this issue, both phenyl and vinyl groups were introduced to synthesize PMPVS, in which phenyl groups can disrupt the regularity of molecular chains to achieve a lower crystallization temperature^[^ ^26^ ^]^ and vinyl groups can reduce the glass transition temperature (*T_g_*). Thus, PMPVS retained a low modulus and elasticity at temperatures as low as −95 °C, which contributed to uniform stress distribution between the gecko‐inspired adhesive and the target surface when in contact and the suction‐cup shape of the mushroom‐shaped arrays upon detachment from the target surface. This resulted in strong adhesion of the PMPVS‐based gecko‐inspired adhesive at extremely low temperatures.

Figure 1e compares the adhesion strength and modulus of the PMPVS‐based gecko‐inspired adhesive with those of adhesives based on zwitterionic hydrogel at −40 °C,^[^ ^27^ ^]^ poly(methyl‐phenyl)siloxane (PMPS) at −120 °C,^[^ ^16^ ^]^ and conventional PDMS at −70 °C. At −40 °C, the PMPVS‐based adhesive exhibited a higher modulus (67.6 kPa) than the hydrogel adhesive (5.7 kPa),^[^ ^27^ ^]^ but a comparable adhesion strength (21.7 kPa). Note that −40 °C was almost the lowest temperature for hydrogel, while PMPVS still owned good adhesion performance at lower temperatures. At −70 °C, compared with the PDMS‐based adhesive, the adhesion strength of the PMPVS‐based adhesive increased by approximately 3600%, while its modulus decreased by 76.4%. Furthermore, at −120 °C, compared with the poly(methyl‐phenyl)siloxane‐based (PMPS) adhesive,^[^ ^16^ ^]^ the PMPVS‐based adhesive exhibited a 245.5% higher adhesion strength. It indicated a remarkable improvement in adhesion performance of the PMPVS‐based adhesive at −70 °C and even at −120 °C. This can be ascribed to the lower modulus at low temperatures of PMPVS introduced with both diphenyl and vinyl groups compared to PMPS modified with monophenyl groups. Consequently, attaching the PMPVS‐based adhesive with a diameter of 1 cm on the fingertip of a cryogenic glove, it can grasp a glass bottle filled with liquid nitrogen (20 g) at −92 °C (Figure 1f; Figure S5, Supporting Information), demonstrating the potential of the PMPVS‐based adhesive for application in extremely low‐temperature environments. This can provide an effective method to apply gecko‐inspired adhesives in helping grippers grasp uncooperative objects in low‐temperature environments (Figure 1g).

**Figure 2:**
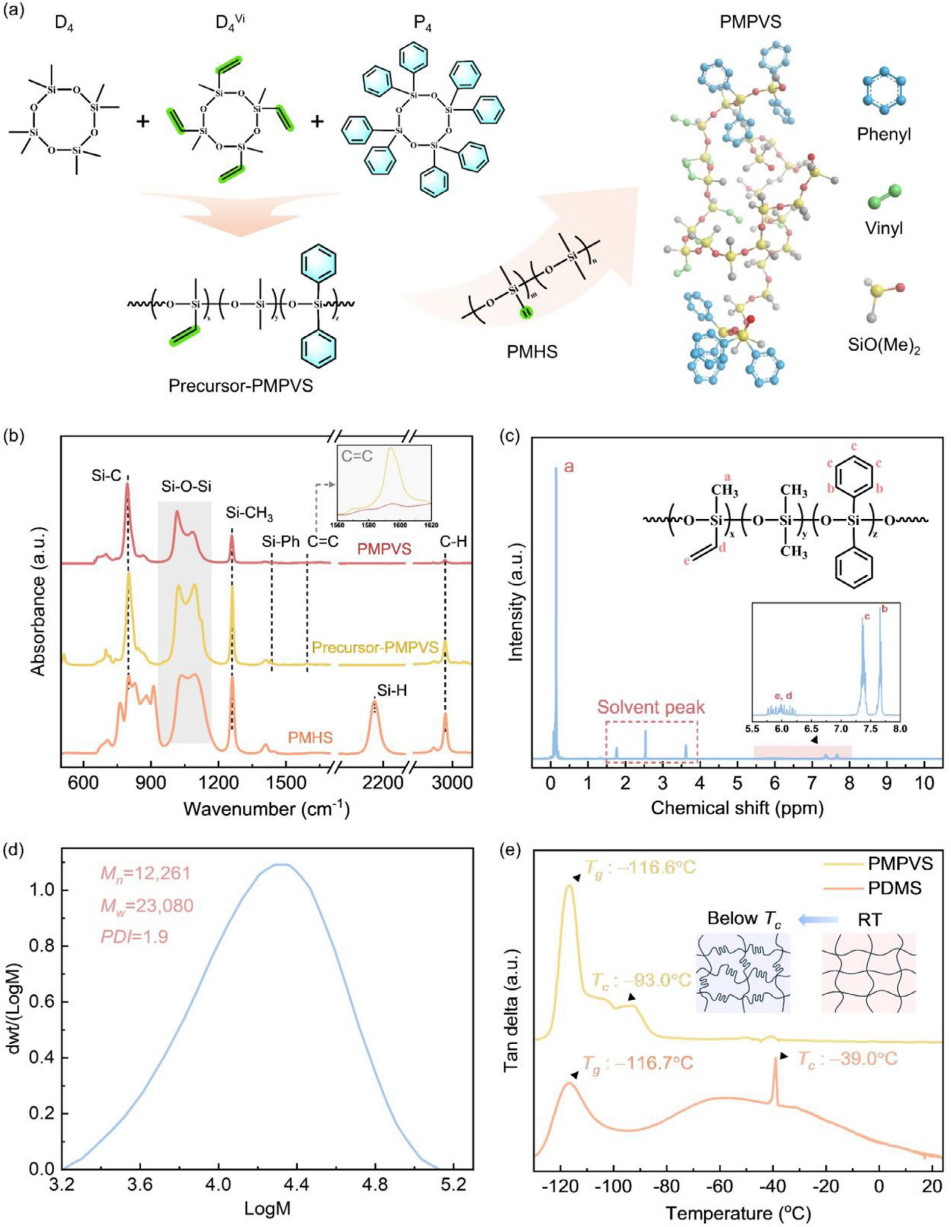
Molecular structures and characteristics of PMPVS. a) Synthesis route and schematic of the molecular structure. b) FTIR spectra. c) ^1^H‐NMR spectrum. d) Differential relative molecular mass distribution spectrum. e) Loss factor curves from −130 to 20 °C.

**Figure** **2** illustrates the preparation and molecular structures of PMPVS. PMPVS was cured through synthesizing precursor‐PMPVS from octamethylcyclotetrasiloxane (D_4_), octaphenylcyclotetrasiloxane (P_4_), and tetramethyltetravinylsiloxane (D_4_^vi^) via anionic ring‐opening polymerization catalyzed by an alkaline gel, with the crosslinking agent poly(methylhydrosiloxane) (PMHS) (Figure 2a). Fourier‐transform infrared spectra (FTIR) (Figure 2b) show that PMHS, precursor‐PMPVS, and PMPVS share peaks at 802 cm^−1^ (Si─C on Si─(CH_3_)_2_), 1024 and 1088 cm^−1^ (Si─O─Si), 1261 cm^−1^ (─CH_3_ on (CH_3_)_2_SiO), and 2962 cm^−1^ (─CH_3_), which indicate the presence of a polysiloxane backbone with methyl groups. For precursor‐PMPVS, peaks at 1429 cm^−1^ (Si─C on Si–phenyl) and 1593 cm^−1^ (C═C on vinyl and phenyl) indicated the presence of phenyl and vinyl groups. Thus, methyl, vinyl, and phenyl groups are present for precursor‐PMPVS.^[^ ^28^ ^]^ The successful synthesis of PMPVS from precursor‐PMPVS and PMHS was verified by the disappearance of the peak of Si─H at 2162 cm^−1^ on PMHS and the greatly reduced intensity of the peak of C═C on precursor‐PMPVS at 1593 cm^−1^ (Insets in Figure 2b). The decreased peak intensity at 1429 cm^−1^ can be ascribed to the reduced content of the phenyl ring in PMPVS owing to curing by PMHS. Moreover, the proton nuclear magnetic resonance (^1^H‐NMR) spectrum of precursor‐PMPVS (Figure 2c) shows peaks at 0–0.3 ppm (methyl groups), 7.2–7.5 and 7.5–7.7 ppm (phenyl groups), and 5.6–6.3 ppm (vinyl groups).^[^ ^29^ ^]^ Correspondingly, the characteristic peak area of the vinyl and phenyl groups was calculated to be the content of 5.5 and 5.0 mol%, respectively. The number‐average molecular weight (*M_n_*), mass‐average molecular weight (*M_w_*), and polydispersity index (*PDI*) of precursor‐PMPVS were 12261, 23080, and 1.9 g mol^−1^, respectively (Figure 2d). The glass transition temperatures (*T_g_*) of PMPVS and PDMS were −116.6 and −116.7 °C, respectively, obtained from the first peak of the loss factor curves (Figure 2e)^[^ ^30^ ^]^. Similar *T_g_* for PMPVS and PDMS can be attributed to the tradeoff between the decrease in *T_g_* induced by vinyl groups and the increase in *T_g_* induced by phenyl groups. The crystallization temperatures (*T_c_*) of PMPVS and PDMS were −93.0 and −39.0 °C, respectively, obtained from the second peak on the loss factor curves.^[^ ^30^ ^]^ Note that a peak of the loss factor near −39.0 °C appeares for PMPVS, which can be ascribed to the partial crystallization of dimethylsiloxane segments.^[^ ^31^ ^]^ Thus, in comparison with common PDMS, PMPVS had a similar *T_g_* but a much lower *T_c_*, which can facilitate the resistance to low temperatures.

### 2.2 Low‐Temperature Resistance

**Figure 3:**
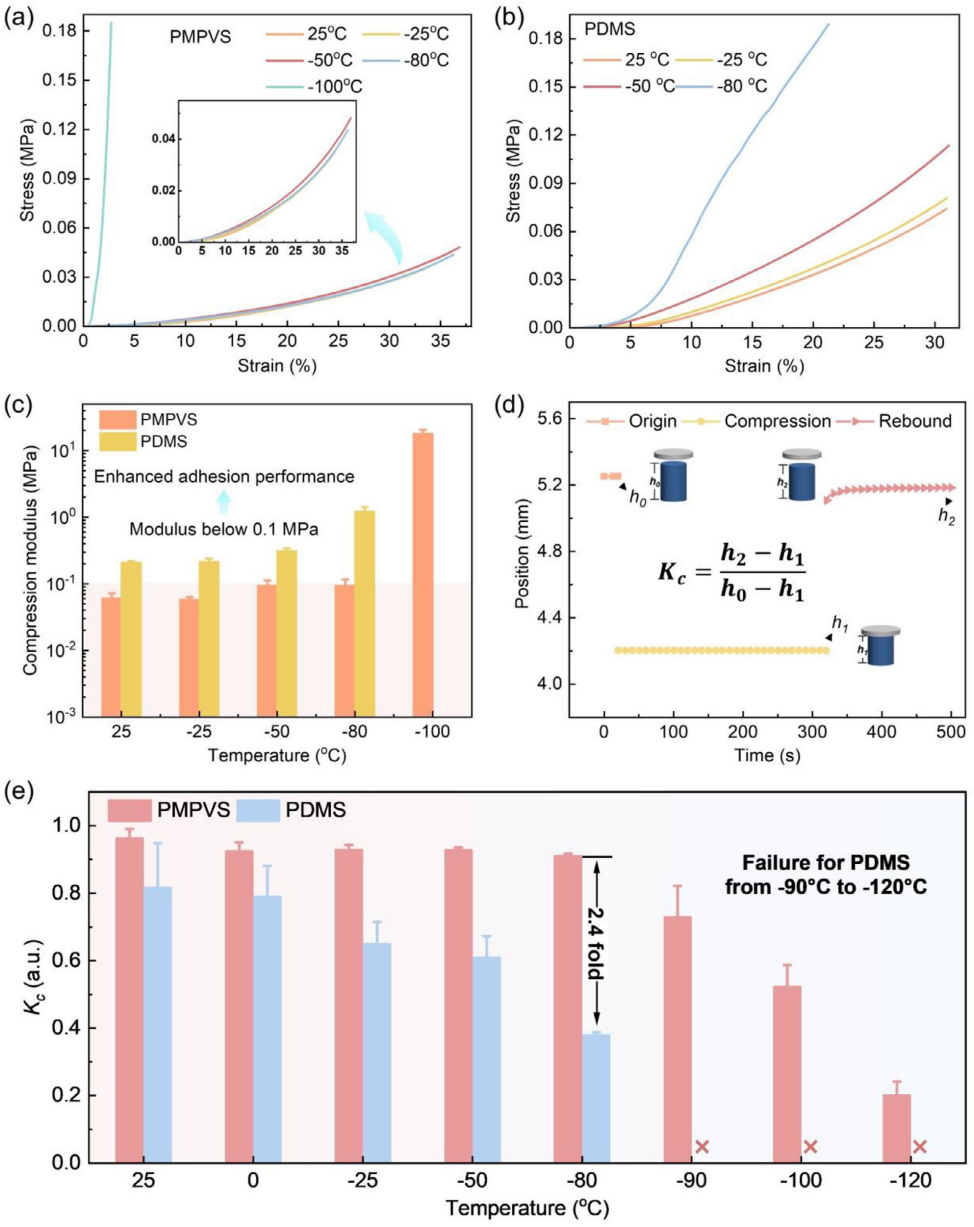
Low‐temperature resistance performances of PMPVS and PDMS. Compression stress–strain curves of a) PMPVS at 25, −25, −50, −80, and −100 °C and b) PDMS at 25, −25, −50, and −80 °C. c) Compression modulus of PMPVS and PDMS. d) Measurement of the compression cold‐resistance coefficient (*K_c_*). e) *K*_c_ of PMPVS and PDMS at different temperatures.

For the gecko‐inspired adhesive, a normal compressive stress is usually applied to enhance the conformal contact with the target surface during the attachment process, in which the compression properties are vital for the adhesion performance.^[^ ^32^ ^]^ **Figure** **3** compares the low‐temperature resistance performances of PMPVS and PDMS. The compression modulus of PMPVS and PDMS (Figure 3c) were obtained from the linear slopes of their compressive stress–strain curves at 10% strain (Figure 3a,b). For PDMS, the compression modulus increased slightly by 2.5% from 25 to −25 °C, and increased greatly to 1.2 MPa and by 468.9% from −25 to −80 °C, which can attribute to the increased crystallinity below *T_c_*. For PMPVS, the compression modulus remained stable from 25 to −25 °C and increased to 94.0 kPa by 63.4% at −80 °C. Thus, at −80 °C, PMPVS displayed a much smaller compression modulus (94.0 kPa) than PDMS (1.2 MPa), which can be an effective modulus of less than 100 kPa according to Kim et al.^[^ ^33^ ^]^ Furthermore, when the temperature was reduced to −100 °C, the compression modulus of PMPVS increased greatly to 18.1 MPa owing to crystallization below *T_c_*. Overall, at low temperatures, PMPVS exhibited much lower compression modulus than PDMS, which facilitated better conformal contact with the target surface and thus excellent adhesion performance.

The recovery capabilities of PMPVS and PDMS are crucial for the low‐temperature resistance of gecko‐inspired adhesives, which were evaluated in terms of the compression cold‐resistance coefficient (*K_c_*, defined as the ratio of the changes in height after recovery to compression) (Figure 3d,e). With decreasing temperature, the *K_c_* of both PMPVS and PDMS decreased due to increased crystallinity. However, PMPVS retained a much higher *K_c_* than PDMS from −80 to 25 °C. At −80 °C, PMPVS exhibited the *K_c_* of 0.91, which was improved by 139.5% as compared to that of PDMS (0.38). Correspondingly, PDMS almost lost its resilience while PMPVS still maintained excellent resilience. Furthermore, PDMS completely lost resilience below −90 °C, whereas the *K_c_* of PMPVS was 0.73 and 0.38 at −90 and −100 °C, respectively. Even at −120 °C, PMPVS had a *K_c_* of 0.20, which indicated that a degree of resilience was retained. The decrease in *K_c_* with the decrease of temperature can be attributed to increased modulus induced by the crystallization below *T*_c_. These results demonstrated the excellent resilience of PMPVS over a wide temperature range and confirm its suitability for application in low‐temperature environments.

### 2.3 Adhesion Performance and Mechanisms

**Figure 4:**
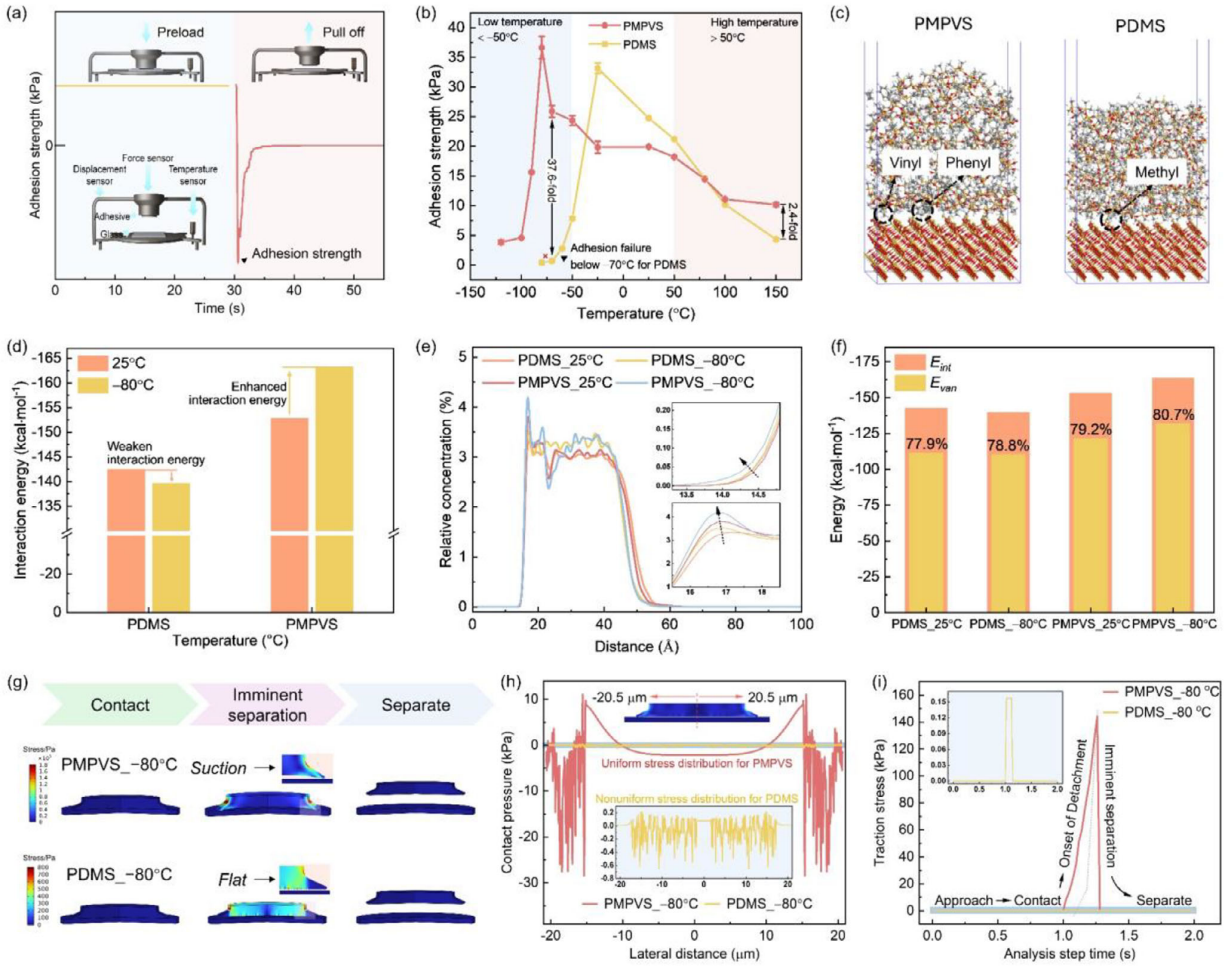
Adhesion performance and mechanism of PDMS‐ and PMPVS‐based adhesives at low temperatures. a) Testing equipment, processes, and adhesion test curve. b) Adhesion strength on glass from −120 to 150 °C. c) Contact between adhesives (PMPVS and PDMS) and SiO_2_ at −80 °C from the molecular dynamic (MD) simulations. d) Comparison of interaction energy at −80 and 25 °C. e) Relative concentration along the *z* (vertical) direction averaged over the *x* and *y* directions on SiO_2_ at different temperatures from the MD simulations. f) Proportion of the van der Waals interaction energy (*E_van_*) in the interaction energy (*E_int_*) at −80 and 25 °C from the MD simulations. g) Dynamic behavior of adhesives when contacting and separating from the surface of the glass at −80 °C and the cloud atlas representing internal stress. h) Contact pressure at the interface and the magnified inset showing the contact pressure of PDMS. i) Traction stress as a function of the processing time.

**Figure** **4** shows the adhesion performances of PMPVS‐ and PDMS‐based gecko‐inspired adhesives. A compression apparatus on a dynamic mechanical analysis (DMA) comprising upper and lower fixtures was used with a temperature sensor, in which the upper fixture was equipped with pressure and displacement sensors to monitor the displacement and force in real time (Figure 4a). The adhesion strength was defined as the ratio of the maximum detachment force of the adhesive from a glass surface to the sample area (Figure 4b). For the PDMS‐based adhesive, the adhesion strength was 24.7 kPa at 25 °C, increased to 33.2 kPa at −25 °C, and decreased greatly to only 0.7 kPa at −70 °C. For the PMPVS‐based adhesive, the adhesion strength was 19.9 kPa at 25 °C, increased to 36.7 kPa at −80 °C, and decreased to 4.6 and 3.8 kPa at −100 and −120 °C, respectively. In comparison with the PDMS‐based adhesive, the PMPVS‐based adhesive retained a much higher adhesion strength. Note that the rate of increase in adhesion strength of the PMPVS‐based adhesive from 150 to 25 °C was lower, which was related to the modulus change at high temperatures (Figure S6, Supporting Information). Notably, compared to PDMS‐based adhesive at −70 °C, the adhesion strength of PMPVS‐based adhesive enhanced by 5142.9% higher at −80 °C and by 442.9% even at −120 °C. Moreover, the adhesion strength of the PMPVS‐based adhesive decreased with increasing surface roughness due to the limited adaptability on rough surfaces but increased with increasing detachment rate due to the reduced degree of deformation of PMPVS, as shown in Figures S7 and S8 (Supporting Information). However, in this work, the adhesion performance of the PMPVS‐based adhesive focused on a relatively smooth glass. Thus, the PMPVS‐based adhesive retains excellent adhesion performance on a relatively smooth glass in low‐temperature environments.

The adhesion strength of polymer adhesives with target surfaces is closely related to the modulus of the adhesives, which plays a key role in conformal contact with target surfaces, interfacial interactions, the stress distribution of the surface for the adhesive, and the formation of the suction‐cup structure. As shown in Figure 3c, at −80 °C, the modulus was as high as 1224.0 kPa for PDMS but moderately low to 94.0 kPa for PMPVS. Correspondingly, PDMS with high modulus cannot form good conformal contact with target surfaces and showed stress concentration, leading to poor adhesion performance. However, at −80 °C, PMVPS with moderately low modulus enabled good conformal contact with the target surface and thus strong interfacial interactions, and showed uniform stress distribution and formed a suction‐cup structure during detachment. As a result, the adhesion strength of the PMPVS‐based adhesive reached 36.7 kPa at −80 °C, improved by 9075% compared with that of the PDMS‐based adhesive (0.4 kPa) at −80 °C.

Furthermore, molecular dynamics (MD) simulations are employed to explore stronger interfacial interactions between PMPVS‐based adhesives and glass representing silicon dioxide (SiO_2_). Simulation detail was found in supporting information. Figure 4c and d presents the interactions between molecular chains of gecko‐inspired adhesives and target surfaces and their interfacial interaction energy (*E_int_*) at 25 and −80 °C. It is found that *E_int_* between PDMS and SiO_2_ increased from −142.5 to −139.6 kcal mol^−1^ from 25 to −80 °C, caused by the decreased chain motility of PDMS at temperatures below *T_c_*. In contrast, *E_int_* between PMPVS and SiO_2_ decreased from −152.8 to −163.3 kcal mol^−1^, indicating enhanced *E_int_* at extremely low temperatures. Moreover, *E_int_* between PMPVS and SiO_2_ at −80 °C was 17.0% lower than that between PDMS and SiO_2_ at −80 °C, demonstrating the superior interfacial interactions of PMPVS with SiO_2_ at extremely low temperatures. These results showed that the adhesion strength decreased for PDMS but increased for PMPVS from 25 to −80 °C. Furthermore, Figure 4e shows the relative concentration of PMPVS and PDMS at 25 and −80 °C along the *z* position. With the decrease of temperature, the relative concentration of PMPVS on the surface of SiO_2_ at around 14.0 and 16.8 Å increased, respectively, and the concentration peak shifted from around 16.8 Å to a slightly smaller distance, demonstrating the closer intermolecular distance and stronger *E_int_* between PMPVS and SiO_2_. Moreover, the *E_int_* is a sum of electrostatic interaction energy (*E_ele_*) and the van der Waals interaction energy (*E_van_*) (Figure S9, Supporting Information). The contribution of *E_van_* was more than 77.9% for both PMPVS and PDMS at both 25 and −80 °C (Figure 4f), which was dominant at the interface.

Moreover, FEA with an air domain is used to probe the stress distribution of the surface and the formation of the suction‐cup structure of the adhesives on a glass surface at −80 °C (Figure 4g; Figure S9, Supporting Information). For the PDMS‐based adhesive, it experienced minimal deformation during separation owing to a sharply increased modulus induced by crystallization below *T_c_*, reducing adhesion strength. For the PMPVS‐based adhesive, detachment initiated from the center of the tips of the mushroom‐shaped array, then the adhesion force caused the tips to deform into a suction‐cup structure with a central groove, and it eventually completely separated from the glass surface. In comparison with the suction‐cup structure with a smaller groove of the tips of the mushroom‐shaped array at 25 °C (Figure S10, Supporting Information), the PMPVS‐based adhesive formed a deeper suction‐cup structure at lower temperatures, which were responsible for enhanced adhesion strength. Furthermore, the contact pressure distribution of the gecko‐inspired adhesives on the glass surface is compared at the beginning of the detachment process with the analysis step time of 1.02 s (Figure 4h). The origin is set to the tip center (0 µm), and the tip radius extends to 20.5 µm. For the PDMS‐based adhesive at −80 °C, the contact pressure varied from −0.8 to 0.2 kPa within the range of −20.5 to 20.5 µm, indicating substantial variability in the stress distribution and thus reduced the adhesion strength.^[^ ^36^ ^]^ In contrast, the contact pressure of the PMPVS‐based adhesive was high within the range of 14.8–20.5 µm–−14.8 to −20.5 µm whereas it was lower and uniformly distributed within the range of −14.8–14.8 µm. This confirmed that the PMPVS‐based adhesive preferentially detached from the center but maintained adhesion at the edges. Within the center of the suction‐cup structure (i.e., −14.8 to 14.8 µm), the PMPVS‐based adhesive displayed a more uniform pressure distribution than the PDMS‐based adhesive (Inset in Figure 4h), resulting in superior adhesion strength. The traction stress exerted on the adhesives was observed during contact and detachment from the glass surface at −80 °C (Figure 4i). The maximum traction stress was only 0.2 kPa for the PDMS‐based adhesive but reached up to 197.5 kPa for the PMPVS‐based adhesive. In addition, the PMPVS‐based adhesive required longer separation time at the same attachment and detachment speeds. This can be attributed to greater deformation and deeper grooves for the PMPVS‐based adhesive, resulting in enhanced adhesion strength.

Moreover, to confirm applications of this PMPVS‐based adhesive in space environments, Figure S11a (Supporting Information) shows the adhesion strength of the PMPVS‐based adhesive at 25, 80, and 150 °C in vacuum environments. At 25, 80, and 150 °C, the adhesion strength of the PMPVS‐based adhesive in the vacuum was 14.5, 10.6, and 9.7 kPa, respectively, which decreased by 27.3%, 26.6%, and 4.9%, respectively, compared with these (20.0, 14.5, and 10.2 kPa) in the air. This was mainly due to the suction‐cup structure with no negative pressure for the PMPVS‐based adhesive in the vacuum (Specific FEA in vacuum in Figure S11b–d, Supporting Information). Importantly, the PMPVS‐based adhesive in the vacuum showed considerable adhesion strength, demonstrating its capability to meet the adhesion requirements of space applications. These results confirm the promising potential of the PMPVS‐based adhesive for stable operation in extremely low‐temperature and vacuum environments.

### 2.4 Cycling Performance

**Figure 5:**
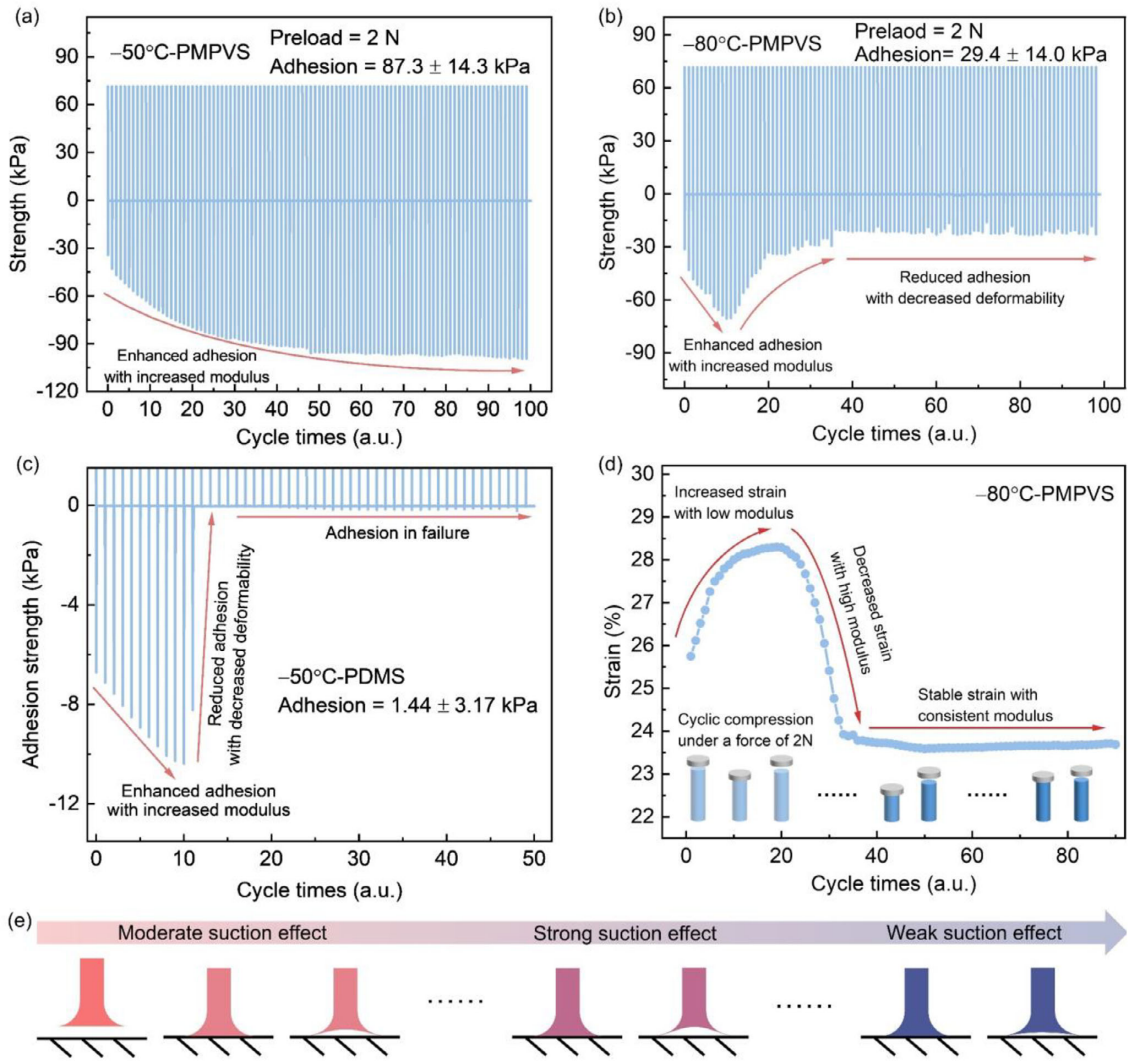
Cycling performances of the gecko‐inspired adhesives under an initial load of 2 N at low temperatures. Adhesion strength of the PMPVS‐based adhesive according to the number of cycles at a) −50 and b) −80 °C. c) Adhesion strength of the PDMS‐based adhesive according to the number of cycles at −50 °C. d) Compressive strain of the PMPVS according to the number of cycles at −80 °C. e) Schematic diagram for the changes in the adhesive during the cycles.

**Figure** **5** shows the adhesion strength of the adhesives as a function of the number of cycles at −50 and −80 °C. For the PDMS‐based adhesive at −50 °C, the average adhesion strength at −50 °C was only 1.44 kPa after 50 cycles. Specifically, the adhesion strength increased from 6.7 kPa for the first cycle to 10.4 kPa for the 11th cycle but decreased sharply to below 0.2 kPa beyond the 13th cycle (Figure 5c). This can be attributed to the increased modulus of PDMS due to crystallization at the temperature below *T_c_* (−39.0 °C). For the PMPVS‐based adhesive at −50 °C, the adhesion strength increased with the number of cycles (Figure 5a). Specifically, the adhesion strength was 34.3 kPa for the first cycle, increased to 99.3 kPa by 189.5% for the 100th cycle, and had an average value of 87.3 kPa after the 100th cycle. Furthermore, at −80 °C, the adhesion performance of the PDMS‐based adhesive was completely lost. However, the adhesion strength of the PMPVS‐based adhesive was 31.3 kPa for the first cycle, increased to 70.3 kPa for the 11th cycle, decreased to 20.1 kPa for the 36th cycle, and stabilized after that (Figure 5b). Compared with the PDMS‐based adhesive at −50 °C, the PMPVS‐based adhesive had 60.6 times the adhesion strength at −80 °C, which demonstrated its superior adhesion performance at low temperatures.

To clarify the mechanism for the cyclic adhesion behavior of PMPVS, compressive strain was observed with the number of cycles (Figure 5d). At −80 °C, under a compressive load of 2 N, the compressive strain was 25.7% at initial, increased to 28.3% after 19 cycles, but decreased to 23.9% after 33 cycles. Corresponding creep behavior was observed for the PMPVS (Figure S12, Supporting Information). This was primarily because of the changes in the modulus of the PMPVS‐based adhesive induced by the variation of crystallization degree during cyclic adhesion under the load of 2 N, which was verified by X‐ray diffraction (Figure S13, Supporting Information). Specifically, at −80 °C, at initial cycles, the increased degree of crystallinity contributed to a slightly increased modulus, which stabilizes the suction‐cup structure of the tips, as shown in Figure 5e, corresponding to enhanced adhesion strength. Then, the modulus increased greatly due to further increased crystallinity degree, which reduces the deformability of the tips and *s* the size of the internal groove in the suction‐cup structure and thus decreases the adhesion strength. At more than 36th cycles, stably crystallized PMPVS leads to further increased modulus, which prevents the tip from deformation and results in a relatively stable adhesion strength. In addition, for adhesion stability at 75 and 150 °C, the average of cyclic adhesion strength was 16.15 ± 0.68 kPa and 10.13 ± 0.68 kPa, respectively, demonstrating the high‐temperature adhesion stability of the PMPVS‐based adhesive (Figure S14, Supporting Information). In summary, the PMPVS‐based adhesive performs reliably at temperatures from −80 to 150 °C, which indicates that it can widen the practical application range of gecko‐inspired adhesives.

### 2.5 Applications

**Figure 6:**
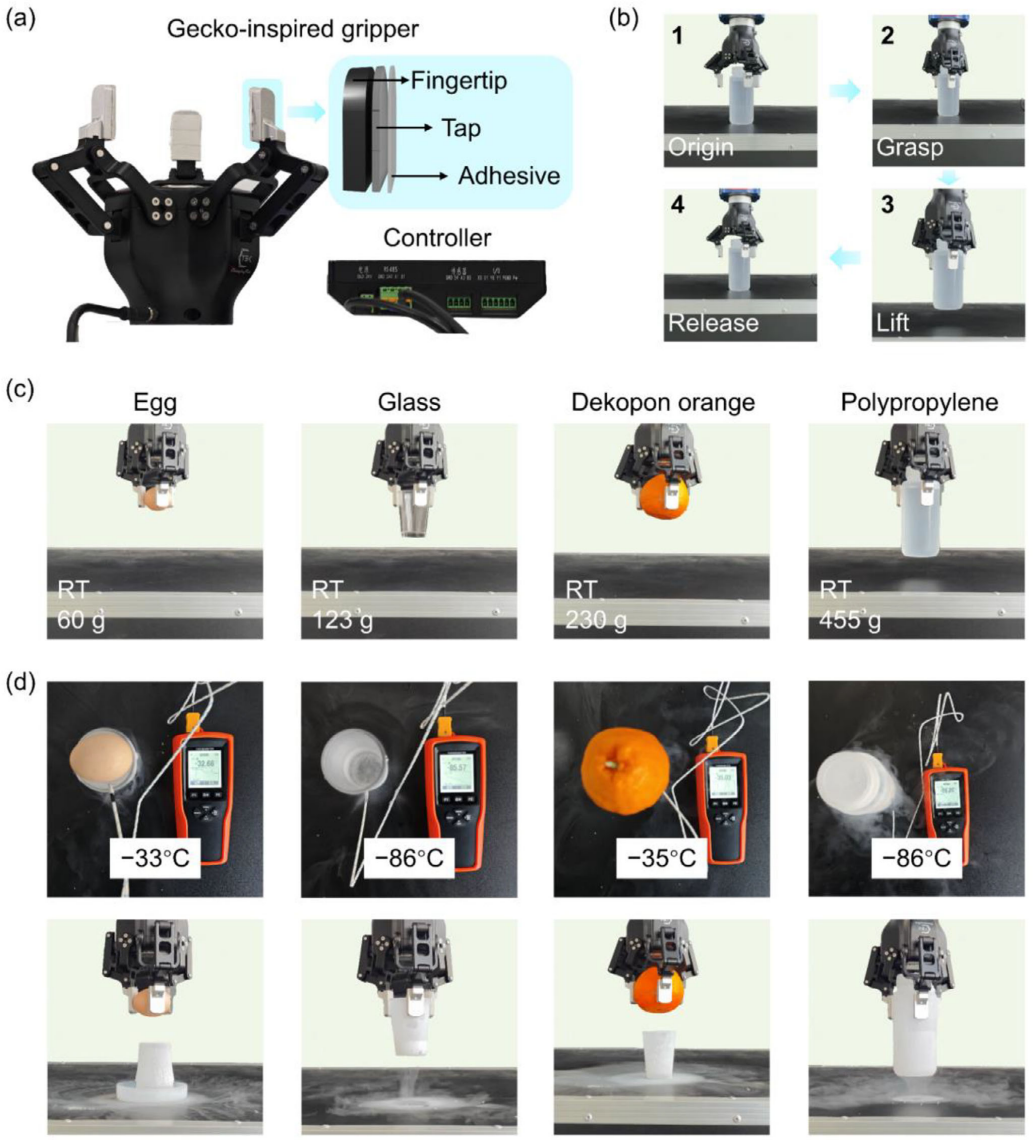
Applications of the PMPVS‐based gecko‐inspired adhesive. a) Schematic of the GiG. b) Grasping process. c) Grasping of the egg, glass, dekopon orange, and polypropylene bottle at room temperature with a gripping force of 2 N. d) Grasping of the egg at −33 °C, glass at −86 °C, dekopon orange at −35 °C, and polypropylene bottle at −86 °C with a gripping force of 2 N.

The above results demonstrated the superior adhesion performance of PMPVS‐based gecko‐inspired adhesive, which exhibits a high adhesion strength, reversible adhesion, and reusability, nondestructive attachment, and residue‐free adhesion. Integrating this adhesive with a traditional robotic gripper can reduce the required gripping force allowing the gripper to gently handle fragile objects, enhance its versatility and reliability, and ensure an efficient and rapid release.^[^ ^10^, ^37^, ^38^ ^]^ **Figure** **6** showed that a gecko‐inspired gripper (GiG) is fabricated by attaching the PMPVS‐based adhesive to the fingertips of a robotic gripper using 3 M double‐sided tape (Figure 6a). The GiG was mounted on a robotic arm, which in turn was controlled by a controller to manage the grasping and releasing actions of three fingers as well as the applied pressure. The target object was placed at a fixed position above the platform (Figure 6b), and the GiG was controlled to grasp the object with a force of 2 N, lift it, and then release it at original position. Each grasping action was repeated at least three times to evaluate the repeatability (Movie S1, Supporting Information). The GiG could grasp various target objects with different weights, shapes, and materials (Figure 6c). With a gripping force of 2 N at room temperature, the GiG stably grasped an egg (60 g), a glass cup (123 g), a dekopon orange (230 g), and a polypropylene plastic bottle filled with water (455 g). Moreover, the GiG could be controlled to transfer target objects to desired locations and release them safely without contamination (Movies S1–‐S3, Supporting Information). Thus, the GiG could grasp fragile, rough, and irregular objects without leaving behind residue. To illustrate the advantages of the low grasping force required by the GiG, it was compared with that of a traditional robotic gripper without adhesives. At a gripping force of 2 N, the traditional gripper failed to grasp the dekopon orange and water‐filled polypropylene bottle, which caused the objects to slip. The traditional gripper required gripping forces of 5 and 20 N to grasp the dekopon orange and water‐filled polypropylene bottle, respectively (Movie S2, Supporting Information). It demonstrated that GiG could reduce the required gripping force by up to 90% compared with the traditional robotic gripper. For comparison, Song et al.^[^ ^39^ ^]^ reported an electro‐adhesive film hybrid gripper that required a gripping force of 2.4 N to stably grasp a 500 g object in the no‐voltage state and a gripping force of 1.7 N at a voltage of 3 kV. Thus, the GiG effectively reduces energy consumption by reducing the required grasping force.

The grasping performance of the GiG was also evaluated at different temperatures (Figure 6d). The surface temperature of an egg and a dekopon orange was lowered to −33 and −35 °C, respectively, by immersion in a liquid nitrogen container (Figure S15, Supporting Information), and a glass cup and polypropylene bottle were filled with liquid nitrogen to reduce their surface temperature to −86 °C. The GiG could stably grasp all four objects at extremely low temperatures (Movie S4, Supporting Information). For comparison, the grasping behavior of the gripper with the PDMS‐based adhesive and the flat PMPVS adhesive at 25 and −86 °C was investigated (Movies S5 and S6, Supporting Information). Grippers with PDMS‐based adhesive and flat PMPVS adhesive failed to hold the polypropylene bottle filled with liquid nitrogen (134 g) at −86 °C and filled with water (455 g) at 25 °C with the gripping force of 2 N, respectively. Thus, the PMPVS‐based gecko‐inspired adhesive exhibited excellent adhesion performance in low‐temperature environments that not only improves the grasping efficiency of robotic grippers but also reduces potential damage to the target objects.

## 3 Conclusion

The PMPVS‐based gecko‐inspired adhesive modifies traditional PDMS with phenyl and vinyl groups to enhance the adhesion performance at low temperatures. PMPVS shows a low glass transition temperature (*T_g_*) of −116.6 °C and a low crystallization temperature (*T_c_*) of −93.0 °C, resulting in a low compression modulus of 94.0 kPa at −80 °C compared with that of PDMS (1.2 MPa) and a high compression cold‐resistance coefficient (*K_c_*) of 0.91 at −80 °C. Consequently, the adhesion strength of the PMPVS‐based adhesive was 25.9 kPa at −70 °C and 36.7 kPa at −80 °C, which shows improvements of 3600% and 5143%, respectively, compared with that of the PDMS‐based adhesive. This can be attributed to the moderately low modulus of PMPVS, enabling good conformal contact with target surfaces and thus strong interfacial interactions with the uniform stress distribution and formation of a suction‐cup structure during detachment. Moreover, the PMPVS‐based adhesive exhibited a stable adhesion strength of approximately 20.1 kPa at −80 °C over 100 cycles, which confirmed its durability and reusability. Furthermore, the robotic gripper with this PMPVS‐based adhesive could securely grasp fragile, rough, irregular, and heavy objects at low temperatures while reducing the required grasping force by up to 90% compared to the robotic gripper without adhesives.

However, the adhesion performance of gecko‐inspired adhesives with micro‐scale structures typically decreases significantly on rough surfaces. This is primarily due to reduced contact area on rough surfaces compared to smooth ones due to poor surface adaptability, thereby lowering the adhesion strength. To address this issue, nanoscale gecko‐inspired adhesives using PMPVS are proposed, however, challenges still exist: nano‐scale structures require advanced processing equipment and techniques, and the low modulus of PMPVS can cause nano‐scale arrays to collapse. The modulus of PMPVS can be enhanced by the introduction of reinforcing fillers (e.g., silicon dioxide or MQ resin) or the construction of dual‐networks. Using porous anodic aluminum oxide (AAO) with nano‐scale structures as a template, PMPVS‐based gecko‐inspired adhesives with nanoscale features can be fabricated, improving adhesion performance on rough surfaces.

In grasping applications, grippers require not only securely and stably capture of target objects but also release on demand. However, for PMPVS‐based gecko‐inspired adhesives, release requires a peeling force exceeding the adhesion force, limiting switchable adhesion. Future work can fabricate tilted arrays to introduce adhesion anisotropy, enabling controlled switching between adhesion and release by adjusting the gripper's motion direction. Overall, this work introduces the PMPVS‐based gecko‐inspired adhesive as a promising candidate for application in low‐temperature environments such as space debris capture, spacecraft docking, and the handling of irregular, fragile samples in extremely low‐temperature environments.

## 4 Experimental Section

### Materials

Octamethylcyclotetrasiloxane (D_4_), 1,3‐divinyltetramethyldisiloxane (MM), tetramethylammonium hydroxide solution (TMAH, 25% in water), 2,4,6,8‐tetramethyl‐2,4,6,8‐tetravinylcyclotetrasiloxane (D_4_^Vi^), octaphenylcyclotetrasiloxane (P_4_), N,N‐dimethylformamide (DMF), bis[1,3‐bis(2‐ethenyl)‐1,1,3,3‐tetramethyldisiloxane]platinum (karstedt catalyst solution), and 1‐ethynyl‐1‐cyclohexanol were purchased from Aladdin. Precipitated silica (R974) was purchased from Evonik Degussa, and the hydrogen silicone crosslinking agent (PHMS, HD31C) was purchased from Chengdu Guibao Science and Technology Co. The silicon plate JGS‐2 and photoresist AZ P4620 were purchased from Suzhou Research Materials Microtech. Polydimethylsiloxane (SYLGARD 184) was purchased from Dow Corning Corporate.

### Synthesis of PMPVS

First, the tetramethylammonium hydroxide alkali gel catalyst was prepared. D_4_ and TMAH were mixed at a mass ratio of 50:1 and vacuum distilled at 35 °C for 2 h to remove water. Then, the reaction was carried out at 95 °C for 0.5 h in a nitrogen atmosphere to obtain a viscous and transparent tetramethylammonium hydroxide alkali gel catalyst, which was stored in a vacuum environment. Next, poly(methyl‐vinyl‐siloxane) with vinyl terminal groups (precursor‐PMPVS) was synthesized. D_4_ (75.0 g, 94.6 mmol), D_4_^Vi^ (1.5 g, 4.4 mmol), P_4_ (4.13 g, 5.2 mmol), MM (1.2 g, 6.4 mmol), DMF (16.1 g, 220.3 mmol), and tetramethylammonium hydroxide alkali gel catalyst (4.0 g) were mixed and vacuum distilled at 35 °C for 2 h to remove water. Then, the reaction was carried out at 105 °C for 2 h in a nitrogen atmosphere. The resulting product was vacuum distilled at 160 °C for 0.5 h to remove unreacted small molecules and obtain the viscous and transparent precursor‐PMPVS was obtained. The vinyl content was measured according to the GB/T 28610‐2020 standard by using an automatic potentiometric titrator (ZDJ‐4a, Shanghai INESA Scientific Instrument Co, Ltd., China). Finally, PMPVS was obtained by curing precursor‐PMPVS and PHMS with a hydrogen to vinyl ratio of 0.15:1. Then, the inhibitor 1‐ethynyl‐1‐cyclohexanol was added at 2 wt.% of the reactive materials. The content of the Karstedt catalyst solution was 8 ppm, and 1.5 wt.% precipitated silica was added. After mixing to ensure homogeneity, the mixture was pumped under negative vacuum pressure to remove air bubbles and cured in a blast oven at 100 °C for 2 h and then at 120 °C for 1 h.

### Fabrication of the Gecko‐Inspired Adhesives

A photolithography procedure^[^ ^25^ ^]^ was used to fabricate the gecko‐inspired arrays on a URE‐2000B mask aligner (Institute of Optics and Electronics, Chinese Academy of Sciences). First, the AZ P4620 photoresist was uniformly spin‐coated onto a 500‐µm‐thick and 2‐in‐diameter silicon wafer at 400 rpm for 40 s and then at 2000 rpm for 40 s, which was followed by a pre‐bake on a hot plate at 70 °C for 2 h to evaporate the solvent. Photolithography was performed by dual exposure of the wafer to UV light at an intensity of 16.6 W cm^−2^ for 180 s on the front side (coated with AZ P4620) and 4 s on the back side (uncoated). This formed the undercut structures critical to the formation of the mushroom tips. Subsequently, the exposed photoresist was developed in a 0.5 wt.% NaOH solution for 530 s, washed in deionized water, and then dried by an air gun to yield a mold for the mushroom‐shaped array of vertical cylinders and wide flat tips. A mixture of the PMPVS matrix and curing agent was cast into the mold and then placed in a vacuum oven at 40 °C for 0.5 h to remove air. Samples were cured at 100 °C for 2 h and 120 °C for 1 h. Demolding was achieved by immersing the cured PMPVS and silicon wafer in anhydrous ethanol for 24 h, which released the array from the mold to obtain the PMPVS‐based gecko‐inspired adhesive. The PDMS‐based gecko‐inspired adhesive was obtained by the same procedure.

### Characterization

FTIR was conducted by using a Nicolet 6700 spectrometer (Thermo Fisher Scientific, USA) in attenuated total reflection mode. Scans were conducted 32 times over a wavenumber range of 650–4000 cm^−1^ at a resolution of 4 cm^−1^. ^1^H‐NMR spectra of PMPVS were obtained with CDCl_3_ as the solvent (Avance neo 400 MHz, Bruker, Germany). The molecular weight of PMPVS was characterized by high‐performance liquid chromatography (1525, Waters, USA) with tetrahydrofuran (THF) as the solvent. The morphology of the gecko‐inspired adhesive was characterized by scanning electron microscopy (Sigma 300, ZEISS Co., Germany). Before the tests, samples were coated with a gold–palladium alloy by using an SBC‐12 ion sputter coater (KYKY, Beijing) for 60 s. The crystallization temperatures of PMPVS and PDMS were obtained by differential scanning calorimetry (TA Instruments, USA). Samples were sliced into pieces with a mass of 5–10 mg and placed in an aluminum crucible, which was heated from 40 to 100 °C at a rate of 20 ^°^C·min^−1^ and then held at 100 °C for 3 min. The sample was then quickly cooled to −95 °C and kept for 1 min to equilibrate, heated to 20 °C at a rate of 3 °C·min^−1^, and cooled to −95 °C at a rate of 3 °C·min^−1^. The loss factor curves, compression stress–strain curves, and compression cold‐resistance coefficient (*K_c_*) were measured by using a dynamic mechanical analysis (DMA) instrument (850 Discovery, TA Instruments, USA). The glass transition temperatures and crystallization temperatures were measured in tensile mode by using rectangular samples with dimensions of 15 mm × 4 mm × 2 mm. The temperature was increased from −135 to 40 °C at a speed of 5 °C·min^−1^, and the amplitude and frequency were set to 15 µm and 1 Hz, respectively. The compression modulus was characterized in compression mode by using cylindrical samples with a diameter and height of both 10 mm. The samples were first placed at the target temperature for 10 min, and the compression strain was set to 30% with a compression speed of 10 mm·min^−1^ to obtain the compression stress–strain curves. The compression cold‐resistance coefficient (*K_c_*) was obtained in compression mode by using cylindrical samples with a diameter and height of both 10 mm according to the HG/T 3866‐2008 standard (Figure 3d). Each sample was placed in the fixture at the target temperature (25, 0, −25, −50, −70, −80, or −100 °C) and compressed to 80% of its original height (*h_0_*). This was held for 5 min, and the compressed height (*h_1_*) was recorded. The compressive load was subsequently removed, and the samples were allowed to recover for 3 min at the same temperature after which the recovered height (*h_2_*) was recorded. Each sample was measured at least three times. *K_c_* was then calculated as follows:

(1) $$K_{c}=\frac{h_{2}-h_{1}}{h_{0}-h_{1}}$$

### Adhesion and Cycling Performance Measurements

The DMA instrument (850 Discovery) was used to characterize the adhesion strength and cycling performance of the adhesives. For the adhesion strength, the same samples as in the compression experiments were used. The shape of the sample was a circle with a diameter of 10 mm. Each sample was first attached to the upper fixture of the compression clamp by using 3 M double‐sided tape. A square glass piece with a side length of 20 mm was attached to the surface of the lower fixture. After the set temperature was maintained for 10 min, an initial load of 2 N was applied to the sample to ensure contact between the sample and the glass surface. After being held for 30 s, the upper fixture was moved upward at a speed of 12 mm min^−1^, and the minimum force was denoted as the adhesion force. Each sample was measured at least three times. For the cycling performance, the DMA instrument was used in compression mode to test cylindrical samples with a height of 5 mm and a diameter of 10 mm. Samples were mounted and tested in the same manner as for the adhesion strength. The test was repeated 100 times to evaluate the cycling performance. The maximum number of cycles was set to 100 because of the memory limitations of the DMA instrument.

### Computational Simulations

Molecular dynamic (MD) simulations were employed to probe the interaction mechanisms between PMPVS or PDMS and SiO_2_ at 298.15 K (25 °C) and 193.15 K (−80 °C). All MD simulations in this study were carried out using Materials Studio software (BIOVIA Software Inc., USA). We established an ideal model of the interface between the PMPVS or PDMS and SiO_2_ to investigate the atomic level interactions at different temperatures. First, we created a computer model of the PMPVS and PDMS molecule, including the polymerization degree, which was chosen to match that of the molecular weight of PMPVS and PDMS. In addition, we modeled the silica supercell substrate (35 Å × 35 Å × 13 Å) with a unit cell of *α*‐quartz to which a layer of PMPVS or PDMS was added to simulate its adhesion. Finally, a dynamic simulation of the NVT ensemble was carried out for 600 ps at 298.15 K and 193.15 K. The trajectories were saved every 10 ps, and the data between 100 ps and 600 ps were used for calculation and analysis. The electrostatic and van der Waals interactions were solved using Ewald summation and the atom‐based method, respectively. We used the COMPASS II (Condensed‐phase Optimized Molecular Potentials for Atomistic Simulation Studies II)^[^ ^40^ ^]^ force field, which had been widely used to calculate atomic‐level interactions between organic‐inorganic substrate interfaces.^[^ ^41^ ^]^ The strength of the interaction between the organic phase polymer and the inorganic phase substrate was measured by the interaction energy (*E_int_*).^[^ ^42^ ^]^

(2) $$E_{int}=E_{total}-\left(E_{polymer}+E_{silica}\right)$$

where *E_int_* is the interaction energy between the PMPVS or PDMS and SiO_2_, *E_total_* is the total energy of the interfacial simulation, *E_polymer_* is the total energy of PMPVS or PDMS after removing the SiO_2_ from the interface, *E_silica_* is the total energy of SiO_2_ after removing the PMPVS or PDMS from the interface. Here, the *E_int_* was calculated as the subtraction of the interaction energy for the gecko‐inspired adhesives and SiO_2_ without surface atoms from the total bonded and nonbonded interaction energy of all atoms after simulation for 600 ps (Figure S16, Supporting Information). All energies are obtained by performing single point energy calculations based on the COMPASS II force field.

### Finite Element Analysis

Finite element analysis (FEA) was conducted to simulate the adhesive behavior of the PDMS‐ and PMPVS‐based gecko‐inspired adhesives on a glass surface at various temperatures in air and vacuum environments. A model was established with a single vertical pillar (4.5 µm in height and 33.0 µm in diameter) and a mushroom‐shaped cap (44.0 µm in diameter and actual diameter of 41.2 µm after the edges were rounded to prevent stress concentration at the tip). Given that PDMS and PMPVS are hyper‐elastic, the neo‐Hookean model was used to represent their properties. The Lamé parameters (i.e., *λ* and *µ*) used to characterize the elastic properties of materials for the neo‐Hookean model^[^ ^43^ ^]^ were obtained by fitting the compression stress–strain curves at various temperatures (Table S1, Supporting Information). The target glass surface was set as a rigid body. The cohesive zone model was used to describe the adhesion between the gecko‐inspired adhesive and glass surface with a detailed description in the supporting information. The cohesive zone model required inputs of the modulus, tensile strength, and the energy release rate associated with tensile failure, which were obtained by fitting the experimental data and influenced the output adhesion strengths. The center point at the surface of the mushroom tip was set as the origin, and its position was controlled to make the mushroom tip contact the glass surface and achieve adhesion (Figure S3, Supporting Information). Then, separation was induced to obtain the force–displacement graphs and curves.

## Conflict of Interest

The authors declare no conflict of interest.

## Supporting information



Supporting Information

Supplemental Movie 1

Supplemental Movie 2

Supplemental Movie 3

Supplemental Movie 4

Supplemental Movie 5

Supplemental Movie 6

## Data Availability

The data that support the findings of this study are available from the corresponding author upon reasonable request.
