# Supplementary material for: Gecko‐Inspired Adhesive for Robotic Grippers with Excellent Ultra‐Low‐Temperature Adhesion Performance
Source: Adv Sci (Weinh). 2025 Nov 14;13(6):e15084. doi: 10.1002/advs.202515084 (PMC12866697; doi:10.1002/advs.202515084)
Supplement: Supplementary file 1 — Supporting Information [file ADVS-13-e15084-s006.pdf]

# Supporting Information

## Gecko-inspired adhesive for robotic grippers with excellent ultra-low-temperature adhesion performance

*Jiabao Feng, Hong Zhu, Yadong Lv, Qi Yang, Guangxian Li, Miqiu Kong*, Wei Pu**

Jiabao Feng, Miqiu Kong, Wei Pu
School of Aeronautics and Astronautics, Key Laboratory of Advanced Spatial Mechanism and Intelligent Spacecraft, Ministry of Education, National Key Laboratory of Advanced Polymer Materials, Sichuan University, Chengdu 610065, PRC
E-mail: pwei@scu.edu.cn (Wei Pu), miqiukong@scu.edu.cn (Miqiu Kong)

Hong Zhu, Yadong Lv, Qi Yang, Guangxian Li, Miqiu Kong
College of Polymer Science and Engineering, National Key Laboratory of Advanced Polymer Materials, Sichuan University, Chengdu 610065, PRC

## Methods

### Molecular dynamics simulation

The adhesion test was conducted on the surface of glass, which was primarily $SiO_2$. Therefore, we first modeled the $SiO_2$ substrate with the dimension of 35.51 Å × 35.15 Å in *x*-*y* plane. Based on the typical exposed surface of silica crystals in their natural environment, a $SiO_2$ supercell model with a thickness of 13.36 Å was created after cleaving the surface along the (0 0 1) crystal plane, considering 1,176 Si and O atoms in a ratio of 1:2 to simulate α-quartz by molecular dynamics simulation. The *α*-quartz with lattice parameters of $a$ = 4.913 Å, $b$ = 4.913 Å, $c$ = 5.405 Å and $\alpha$ = 90°, $\beta$ = 90°, $\gamma$ = 120° was used to construct the silica substrate model. This interface model was subjected to geometric optimization using a smart algorithm to minimize the energy and stabilize the structure of each monomer. The convergence tolerances for energy and force were $1.0 \times 10^{-4}$ kcal/mol and 0.005 kcal/mol/Å, respectively, in accordance with the fine quality convergence criteria outlined in the Material Studio documentation. After geometry optimization, 4 PMPVS chains of 75 monomers or 11 PDMS chains of 30 monomers were placed on the $SiO_2$ surface, respectively. To control the temperature during the simulation of α-quartz, NVT ($N$ = same number of atoms, $V$ = constant volume, $T$ = temperature in $K$) canonical ensemble was used with a time step of 1 fs and a total simulation time of 600 ps, during which simulation trajectories were recorded every 5000 steps. The running time was long enough for system energy and temperature reaching stable. The temperature was controlled by a Nose-Hoover thermostat. The Ewald scheme and atom-based cutoff method (*i.e.*, a radius of 12.5 Å) were applied to treat electrostatic and van der Waals interactions, respectively. The partial charges of each atom were assigned by the force filed. The relative concentration profiles of PMPVS and PDMS along perpendicular direction were analyzed. The interfaces snapshots were also displayed.

### Finite element analysis

To investigate adhesion behaviors of PDMS- and PMPVS-based adhesives on a glass surface in air and vacuum environments, a finite element analysis (FEA) model based on the cohesive zone theory was established. For FEA in air environments, an air domain was introduced as shown in **Figure S1**. Above the air domain was an entrance with a pressure of 1 atm, and below that was an outlet. For FEA in vacuum environments, the model without additional domains around the adhesive was established. The method of establishing the FEA model is as follows.

*Cohesive zone model*: Due to van der Waals interactions between the gecko-inspired adhesive and glass surface, the cohesive zone model (CZM) was used to describe their adhesion behavior. This model captured the progressive failure of adhesion by defining a traction-separation relationship across the interface. In this study, we focused exclusively on normal stress, neglecting friction and shear stress, as normal forces dominated the adhesion mechanism under normal preload. The traction-separation behavior is depicted in **Figure S2**, which illustrates the relationship between normal traction and interfacial separation. The curve typically rose linearly to a maximum stress, $\sigma_{iC}$, representing the adhesion strength, then decreased as separation increases, reflecting softening and eventual bond failure. The area under this curve was the critical energy release rate, $G_{iC}$, which quantified the energy required for crack propagation. When the applied load exceeded $\sigma_{iC}$, crack propagation initiated, reducing the force capacity as the interface degraded. The specific governing equations are described as follows.

*Governing Equations for Adhesion and Stripping*: The adhesion behavior was modeled using a finite element approach with cohesive elements embedded at the adhesive-glass interface. This damage-based framework accounted for both the elastic response of the adhesive and its degradation under increasing normal loads. The constitutive relation for the cohesive elements is given by:

$$f = (1 - d)ku = ku - dku \tag{1}$$

This equation represents the adhesive force $f$ in terms of the stiffness matrix $k$, the displacement vector $u$, and a damage variable $d$. This equation expresses how the adhesive force decreases as damage accumulates. In the undamaged state ($d$ = 0), the adhesive force is purely elastic, proportional to the displacement. As damage increases ($d$ > 0), the adhesive force decreases, reflecting the loss of adhesion due to the degradation of the material. The damage variable $d$ is defined through a functional relationship involving the maximum displacement and critical fracture energies:

$$d = F_{\dots}^{-1}(u_{\text{max}}, \sigma_{\text{t}}, \sigma_s, G_{\text{ct}}, G_{\text{cs}}) \tag{2}$$

where $F^{-1}$ is an inverse function that determines the damage variable based on the following parameters. The maximum displacement $u_{max}$ experienced by the adhesive element. This condition ensured that the maximum displacement is recorded and used to compute damage, preventing "healing" effects where the material would recover after deformation:

$$u_{\text{max}} = \max\left(u_{\text{max}}, u_{\text{max,old}}\right) \tag{3}$$

$\sigma_t$ and $\sigma_s$ represent the maximum normal and shear stress that the interface can withstand before separation, which is determined by dividing the peak force by the contact area obtained from the force–displacement curve during adhesion testing, respectively. $G_{ct}$ and $G_{cs}$ represent the tensile and shear energy required for the propagation of a unit interfacial crack and can be derived from the integration of the force–displacement curve, respectively. In this work, only normal adhesion was investigated, so the effect of shear ($\sigma_s$ and $G_{cs}$) could be ignored. The $\sigma_t$ of PMPVS and PDMS at −80°C was 37.1 kPa and 1.0 kPa, respectively, and $G_{ct}$ of those at −80°C was 19.7 J·m$^{-2}$ and 3.0 J·m$^{-2}$, respectively. As $\sigma_t$ and $G_{ct}$ increased, the adhesion strength obtained in FEA increased.

The damage evolution is governed by a failure criterion, expressed as:

$$\left(\frac{G_I}{G_{\mathrm{ct}}}\right)^{\alpha} + \left(\frac{G_{II}}{G_{\mathrm{cs}}}\right)^{\alpha} = 1 \tag{4}$$

where $G_I$ and $G_{II}$ represent the energy release rates in the tensile and shear directions, respectively. $\alpha$ is a material-specific exponent that defines the interaction between tensile and shear damage modes.

The criterion ensures that the damage evolves when the energy release rates exceed the critical values $G_{ct}$ and $G_{cs}$, dictating whether the adhesive element will fail in a tensile, shear, or mixed-mode fashion.

*Setting of air domains*: The air domain height and radius were set to 80 μm and 160 μm, respectively. The motion of the air within and around the adhesive was described by the Navier-Stokes equations for an incompressible Newtonian fluid. The governing equations are expressed as:

$$\rho(u_2 \cdot \nabla)u_2 = \nabla \cdot [-pI + K] + F \tag{5}$$

$$\rho\nabla \cdot u_2 = 0 \tag{6}$$

in which $\rho$ is the fluid density, $u$ is the fluid velocity vector, $p$ is the static pressure field within the air domain, $I$ is the indemnity tensor representing isotropic pressure effects, $K$ is the viscous stress tensor, $F$ is external body force vector. Equations (5) and (6) represent the momentum conservation law and incompressibility condition, respectively.

At the interface between the air domain and the adhesive surface, the air exerts a distributed traction that couples into the structural solver. The local force per unit area on the solid surface is given by:

$$F_A = [-pI + K] \cdot n \tag{7}$$

in which $F_A$ is aerodynamic traction vector acting on the solid surface, $n$ is the unit outward normal vector of the solid surface, $pI$ is the isotropic pressure stress, $K$ is the viscous shear stress contribution due to air motion along the surface. Equation (7) establishes the Neumann-type boundary condition for coupling between the fluid and solid domains.

The nonlinear elastic behavior of the gecko-inspired adhesive comprised PMPVS and PDMS was modeled using the Neo-Hookean hyperelastic model. **Table S1** provided material parameters at −80°C. Here, $\lambda$ and $\mu$ are the Lamé constants derived from experimental data, ensuring accurate representation of the adhesive's mechanical response under the simulated conditions.

In the simulation, a piecewise function was applied to control the contact and separation between the adhesive and target surface, as shown in **Figure S3**. The displacement referred to $z$ position of the adhesive. The initial distance between the adhesive and target surface was set to 2 μm. As the displacement decreased from 0 μm to -2 μm with the analysis step time from 0 s to 1 s, the adhesive moved downward by 2 μm, which was equal to the distance between the adhesive and target surface. Thus, at 1 s of the analysis step time, the adhesive contacted the target surface and initiated adhesion. Subsequently, over the next 1 s, the adhesive moved upward by 4 μm, detaching from the target surface.

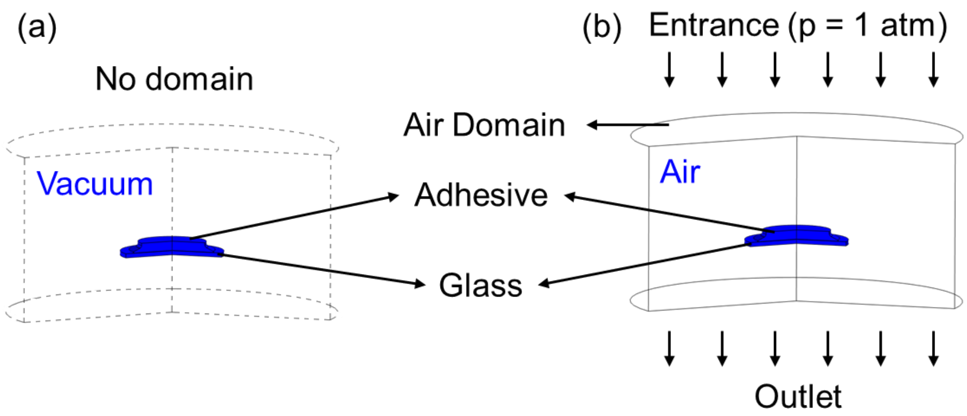


**Figure S1. Schematic of the finite element model in (a) vacuum and (b) air environments.**

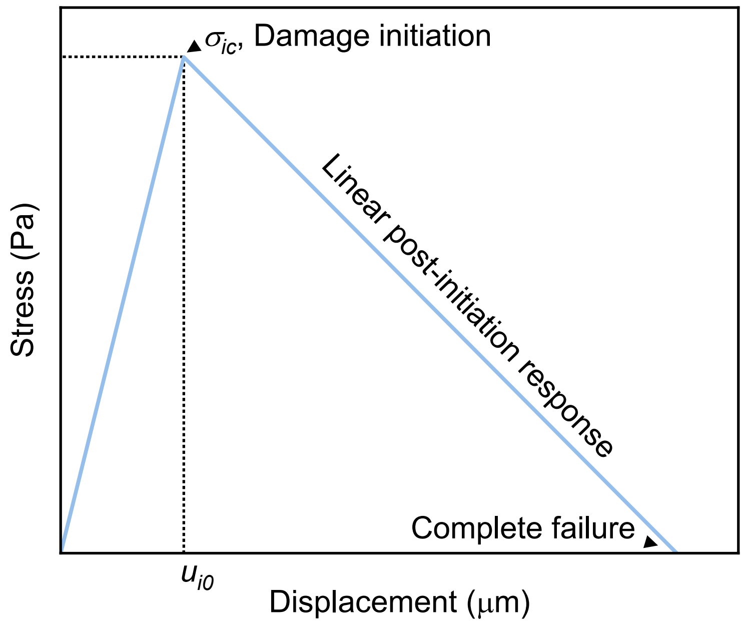


**Figure S2. The relationship between normal stress and boundary separation for the cohesive zone model (CZM).**

**Table S1. The parameters of PMPVS and PDMS for the Neo-Hookean model.**

| Material | Temperature/°C | Parameters/MPa |
| --- | --- | --- |
| PMPVS | -80 | $\lambda = 1.0$, $\mu = 0.71$ |
| PDMS | -80 | $\lambda = 0.5$, $\mu = 9.39$ |

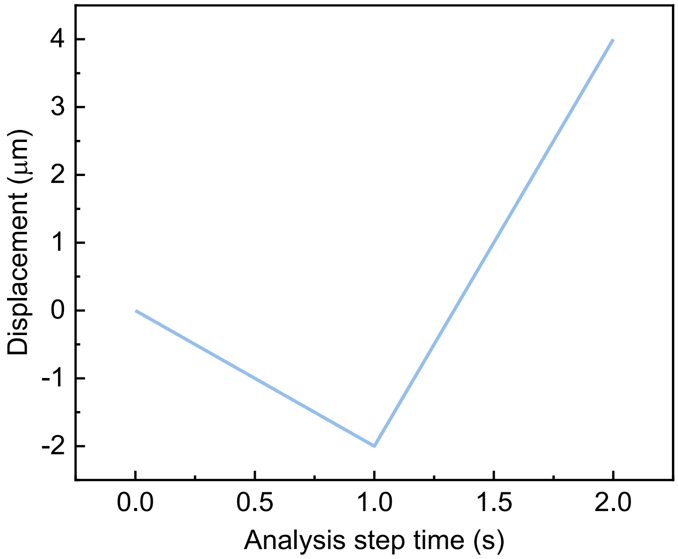


**Figure S3. Displacement control of gecko-inspired adhesives in finite element analysis (FEA).**

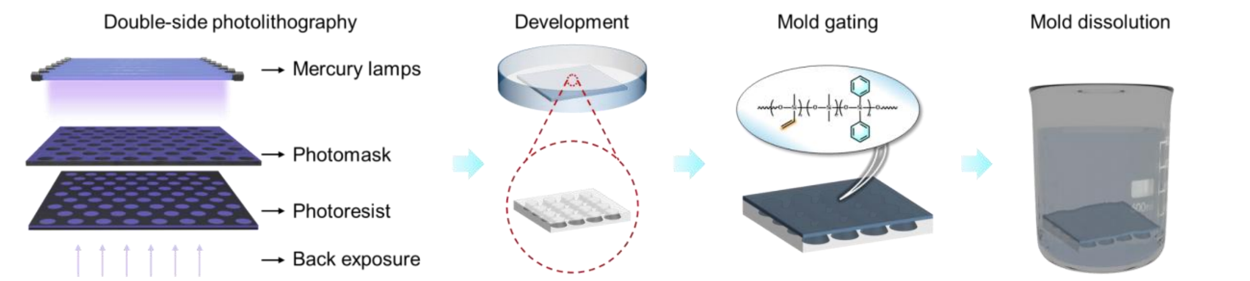


**Figure S4. The fabrication process of gecko-inspired adhesives with mushroom-shaped arrays.**

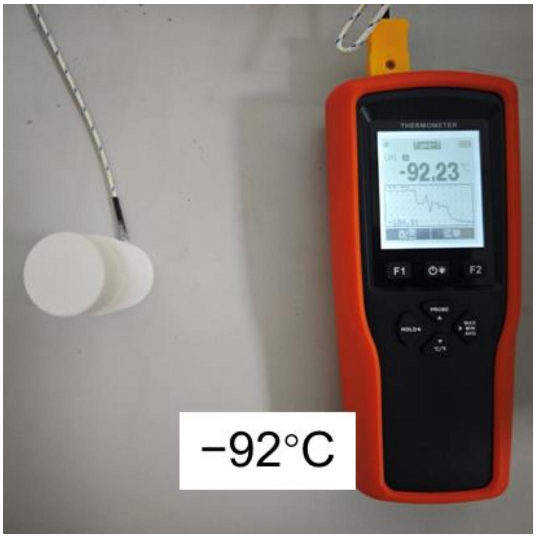


**Figure S5. The temperature at the bottom of the glass bottle filled with liquid nitrogen.**

**Figure S6** shows the storage modulus of PMPVS and PDMS from −120°C to 150°C by using a dynamic mechanical analysis (DMA) with a heating rate of 5°C/min. It can be observed that the storage modulus of PDMS was relatively stable at about 136.0 kPa from 25°C to 150°C, resulting in limited formation of the suction-cup structure and thus a rapid decrease in the adhesion strength due to the weakened intermolecular interactions. However, the storage modulus of PMPVS increased from 149.1 kPa to 171.9 kPa from 25°C to 150°C due to the enhanced entropic elasticity and relaxation of dangling chains that re-engage in the stress-bearing network. With the increase of the temperature, the increased modulus promoted deeper suction-cup structure and thus the adhesion strength increased, but intermolecular interactions decreased and thus the adhesion strength decreased. Both aspects compensated, resulting in lower rate of increase in adhesion strength of the PMPVS-based adhesive from 150°C to 25°C.

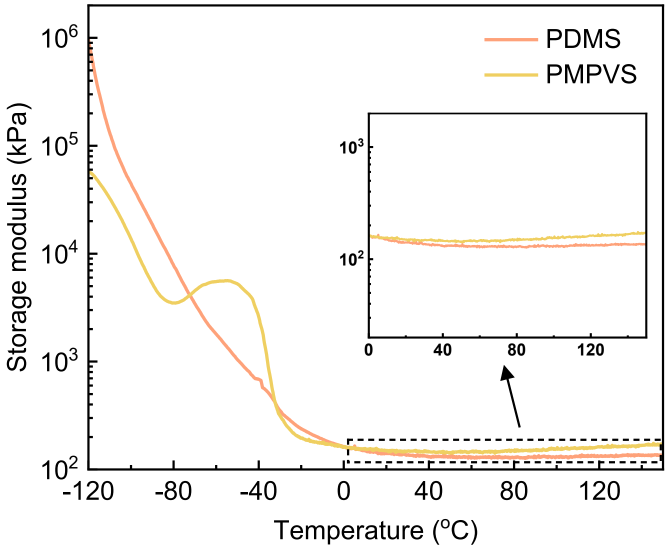


**Figure S6. Storage modulus of PDMS and PMPVS as a function of temperature from −120°C to 150°C.**

**Figure S7** shows the adhesion strength of the PMVPS-based adhesive on the glass with arithmetic mean roughness $R_a$=1.9 μm and 40.8 μm, respectively, and on aluminum (Al) surfaces with $R_a$=7.6 μm and 35.3 μm, respectively, under the preload of 2Nat 25°C, in which $R_a$ is obtained by a laser scanning confocal microscopy (LSM700, ZEISS, German). It is observed that the adhesion strength of the adhesive was 20.8 kPa and 19.6 kPa on surfaces of glass with $R_a$=1.9 μm and Al with $R_a$=7.6 μm, respectively. In contrast, the adhesion strength decreased to 10.6 kPa and 9.0 kPa on surfaces of glass with $R_a$=40.8 μm and Al with $R_a$=35.3 μm, which was decreased by 49.0% and 54.1% as compared to their relatively smooth ones, respectively. This was mainly because with the increase of $R_a$, the adaptability of the adhesive to the target surface decreased, and the effective contact area decreased significantly, which led to a decrease in the adhesion strength.

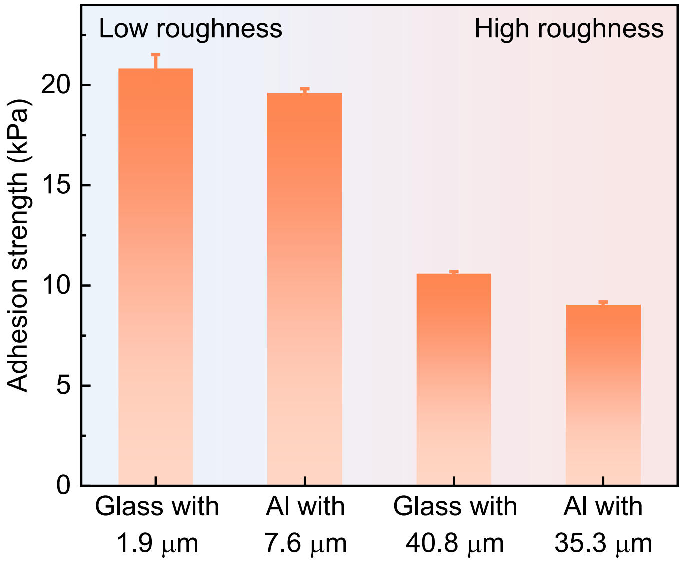


**Figure S7. The adhesion strength of the PMPVS-based adhesive on the glass with $R_a$ = 1.9 and 40.8 μm and the Al with $R_a$ = 7.6 and 35.3 μm, under the preload of 2 N at 25°C, respectively.**

**Figure S8** gives the adhesion strength of the PMPVS-based adhesive at −80°C as a function of the detachment rate. It is observed that the adhesion strength increased with the increase of the detachment rate from 12 mm/min to 100 mm/min. This was mainly because the suction-cup structure was more stable at higher detachment rate due to the reduced degree of deformation of PMPVS since the molecular chain of PMPVS had no sufficient time to rearrange and relax.

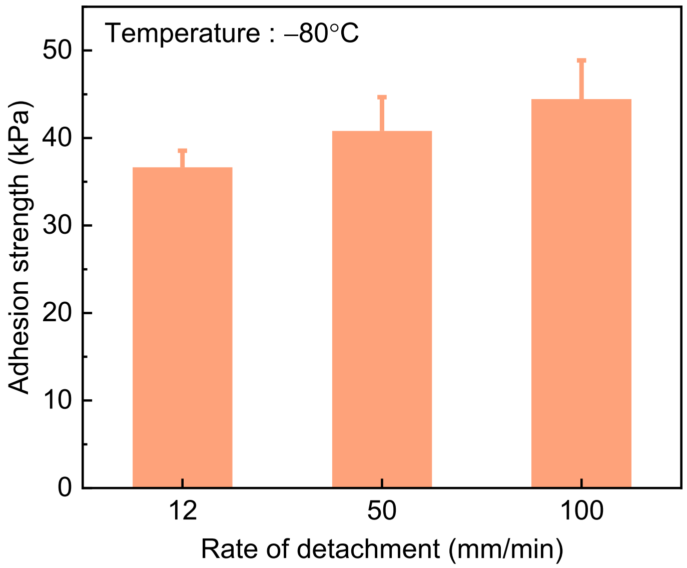


**Figure S8. The adhesion strength of the PMPVS-based adhesive at different detachment rates (12 mm/min, 50 mm/min, and 100 mm/min) at −80°C.**

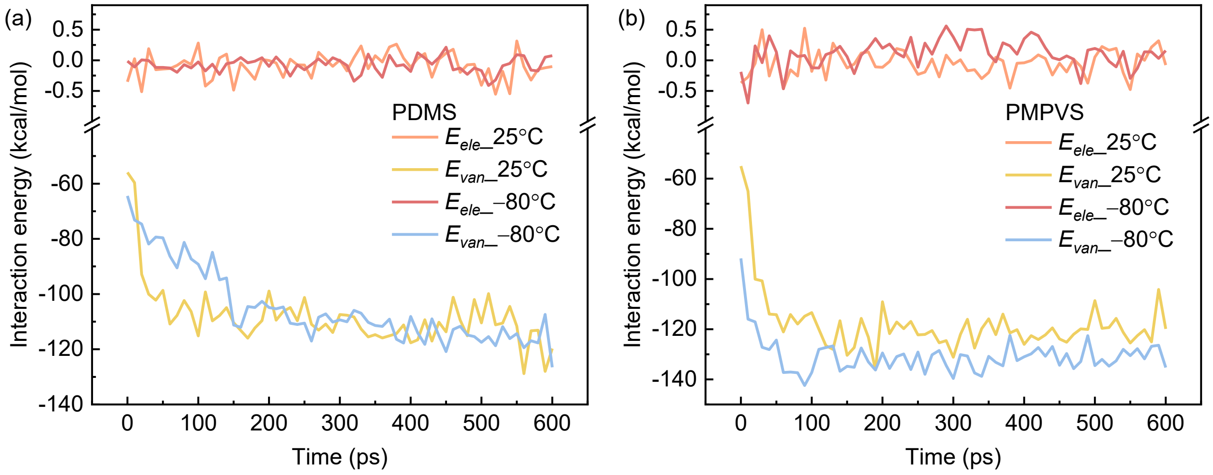


**Figure S9. The electrostatic interaction energy ($E_{ele}$) and the van der Waals interaction energy ($E_{van}$) between (a) PDMS and $SiO_2$ and (b) PMPVS and $SiO_2$ at 25°C and −80°C from 0 ps to 600 ps.**

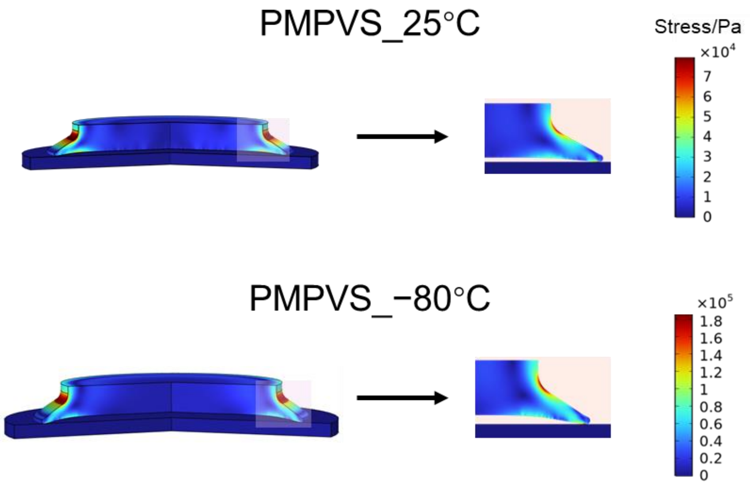


**Figure S10. The detachment process of the PMPVS-based adhesive at 25°C and −80°C, respectively, with the cloud atlas as the internal stress.**

**Figure S11a** shows the adhesion strength of the PMPVS-based adhesive at 25°C, 80°C, and 150°C in air and vacuum environments, in which the adhesion strength in vacuum is conducted on a vacuum universal testing machine (EUT4204, Shenzhen SAAS testing technology co., China). At 25°C, 80°C, and 150°C, the adhesion strength of the PMPVS-based adhesive was 14.5 kPa, 10.6 kPa, and 9.7 kPa in the vacuum, respectively, which decreased by 27.3%, 26.6%, and 4.9%, respectively, compared with these (20.0 kPa, 14.5 kPa, and 10.2 kPa) in the air. This was mainly due to the suction-cup structure with no negative pressure for PMPVS-based adhesive in the vacuum. Note that with the increase of the temperature, the difference between adhesion strength in the vacuum and air diminished, which was attributed to the decreased depth of the suction-cup structure and weakened negative pressure induced by the decreased modulus.

Furthermore, finite element analysis (FEA) was employed to reveal the difference in adhesion behavior between air and vacuum. **Figure S11b** shows the suction-cup structure during detachment from the glass surface for the PMPVS-based adhesive at −80°C in the air and vacuum. However, the internal stress within the adhesive in the air was significantly higher due to the contribution of negative pressure. Moreover, **Figure S11c** presents the interfacial stress distribution between the PMPVS-based adhesive and glass at −80°C in the vacuum and air. The origin is set to the tip center of the PMPVS-based adhesive (0 μm), and the tip radius extends to 20.5 μm. For both vacuum and air, large contact pressure was observed in the regions from 14.8 to 20.5 μm and from −14.8 to −20.5 μm, while uniform and smaller pressure was found within the range from −14.8 to 14.8 μm. This confirmed that the PMPVS-based adhesive preferentially detached from the center but maintained adhesion at the edges. Furthermore, **Figure S11d** shows the variation in traction force during contact and separation processes in the air and vacuum at −80°C. The maximum traction force in the air reached 197.5 kPa, which was approximately 36.9% higher than that in the vacuum (144.3 kPa). This enhancement could be attributed to the negative pressure effect within the suction-cup structure under air conditions. Importantly, the PMPVS-based adhesive in the vacuum showed considerable adhesion strength, demonstrating its capability to meet the adhesion requirements of space applications. These results confirm the promising potential of the PMPVS-based adhesive for stable operation in extremely low-temperature and vacuum environments.

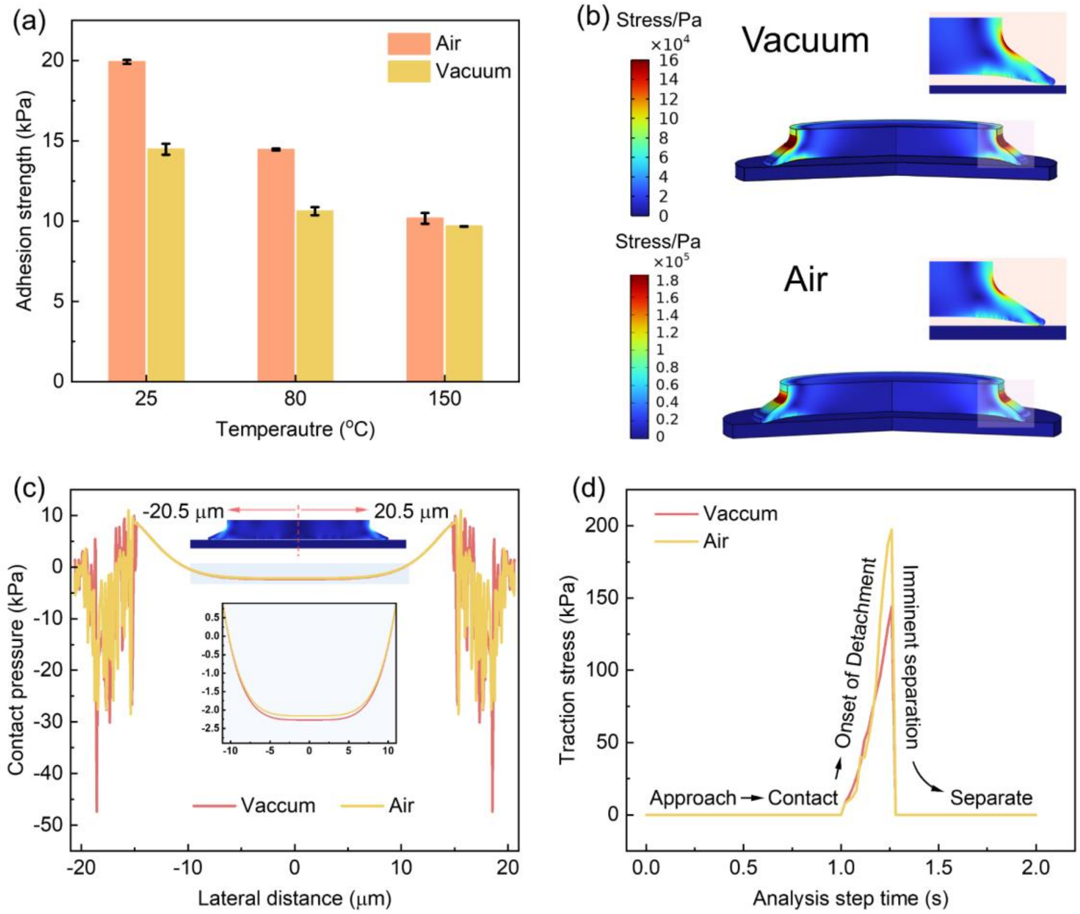


**Figure S11. Comparison of the adhesion behavior of the PMPVS-based adhesive in the vacuum and air.** (a) The adhesion strength of the PMPVS-based adhesive in air and vacuum environments at 25°C, 80°C, and 150°C. (b) Dynamic behavior of the PMPVS-based adhesive when separating from the surface of glass at −80°C and the cloud atlas representing internal stress. (c) Contact pressure at the interface. (d) Traction stress as a function of the processing time.

**Figure S12** illustrates the creep behavior of PMPVS under a preload of 2 N at −80°C for 1 h. It is observed that the creep compliance of PMPVS increased significantly from 17.1 $MPa^{-1}$ to 21.0 $MPa^{-1}$ within 40 min and stabilized around 21.0 $MPa^{-1}$ from 40 min to 60 min. This led to a significantly increased modulus of PMPVS induced by the promoted crystallinity and a relatively stable modulus induced by the stable crystallinity, which was responsible for initially increased, then decreased and stabilized adhesion strength with the cycle time.

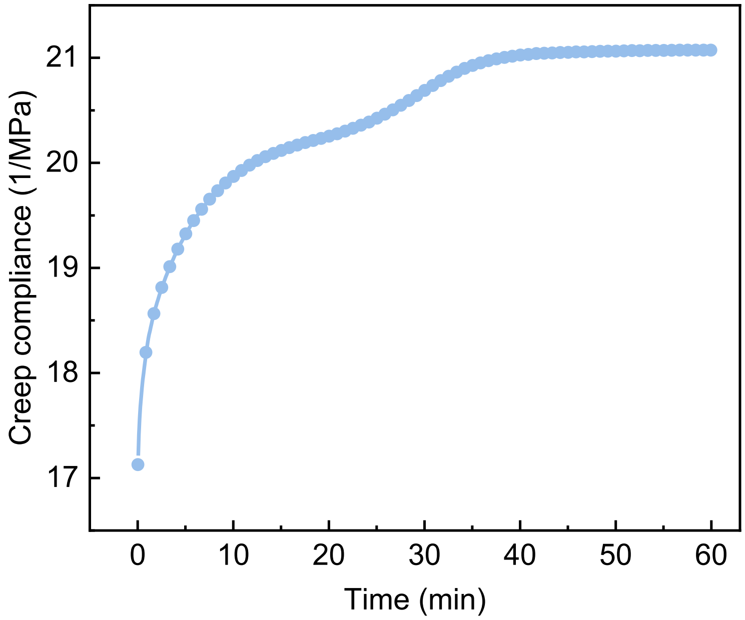


**Figure S12. The creep compliance of PMPVS as a function of the time under a preload of 2 N at −80°C for 1 h.**

X-ray diffraction (XRD) was conducted on a Ultima IV (Rigaku, Japan) to characterize the crystallinity of original PMPVS, PMPVS with cycling adhesion at −80°C for 11 times, 38 times and 100 times under a preload of 2 N, respectively, as shown in **Figure S13a**. Samples were scanned from 5° to 50° at a speed of 5°/min, the shape of which was a cylinder with a diameter and height of 10 mm and 5 mm, respectively. Cu target was used as the X-ray source. For original PMPVS, a scattering peak was detected at approximately $2\theta$ from 25° to 35°, showing obvious amorphous characteristics. For PMPVS with cycling adhesion for 11, 38, and 100 times, the main diffraction peaks were located at $2\theta$=12.3°, $2\theta$=12.3°, and $2\theta$=12.8°, showing obvious semicrystalline characteristics. Furthermore, the degree of crystallinity $\chi_c$ can be calculated by the multi-Gaussian fitting result in the software Jade:

$$\chi_c = \frac{A_c}{A_c + A_a} \tag{8}$$

in which, $A_c$ represents the area of crystalline peaks of diffraction, and $A_a$ represents the area of amorphous peaks of diffraction. **Figure S13b** shows their degree of crystallinity $\chi_c$. It was found that the crystallinity was only 2.5% for original PMPVS and increased to 9.14%, 32.4%, and 32.5% for PMPVS for 11$^{th}$, 38$^{th}$, and 100$^{th}$ cycle. This resulted in the increased modulus of PMPVS.

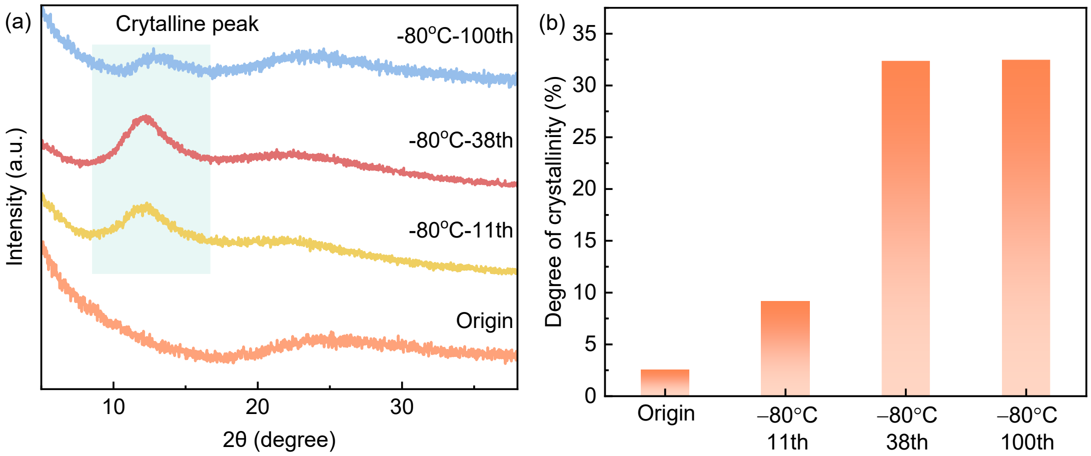


**Figure S13. (a) X-ray diffraction curves and (b) corresponding degree of crystallinity for original PMPVS, PMPVS with cycling adhesion at −80°C for 11$^{th}$, 38$^{th}$, and 100$^{th}$ cycle under a preload of 2 N.**

**Figure S14** gives the adhesion strength of the PMPVS-based adhesive for 100 adhesion cycles at 75°C and 150°C. At 75°C and 150°C, the average of cyclic adhesion strength was 16.15±0.68 kPa and 10.13±0.68 kPa, respectively. This indicated that the adhesion performance of the PMPVS-based adhesive remained relatively stable during high-temperature cycling adhesion due to relatively stable modulus at high temperatures.

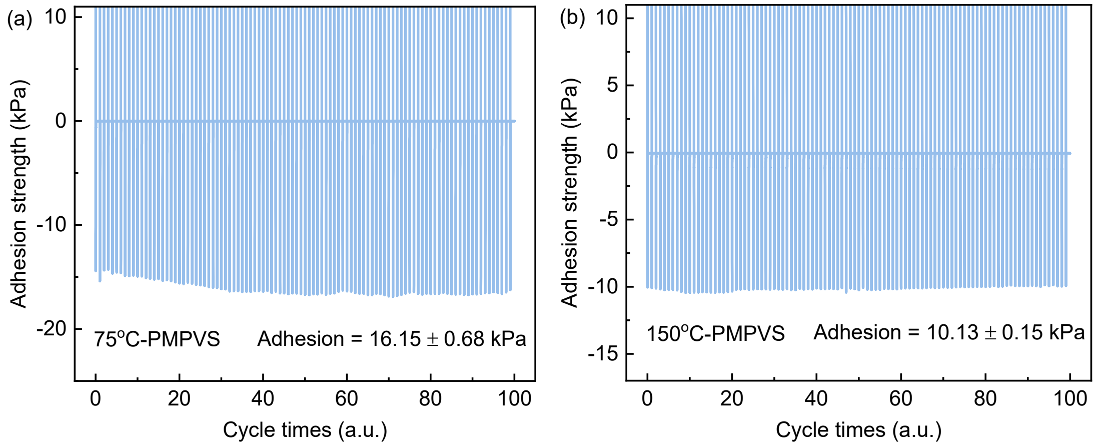


**Figure S14. Adhesion strength of the PMPVS-based adhesive as a function of cycle times at (a) 75°C and (b) 150°C, respectively.**

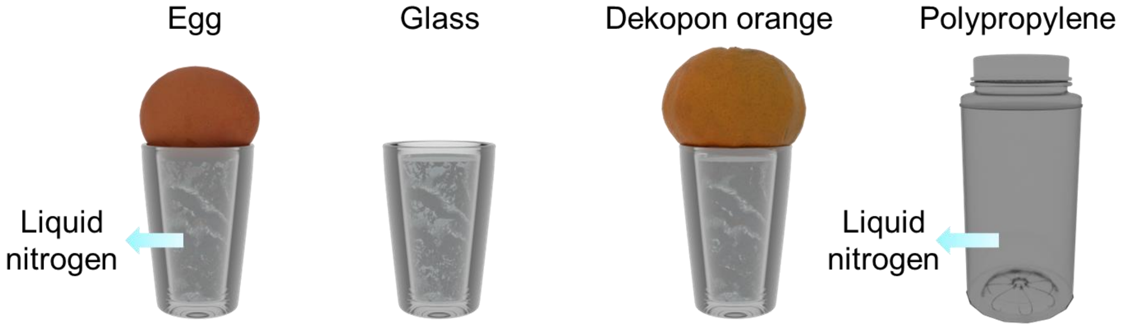


**Figure S15. Schematic diagram of liquid nitrogen cooling of the egg, glass, dekopon orange and polypropylene bottle.**

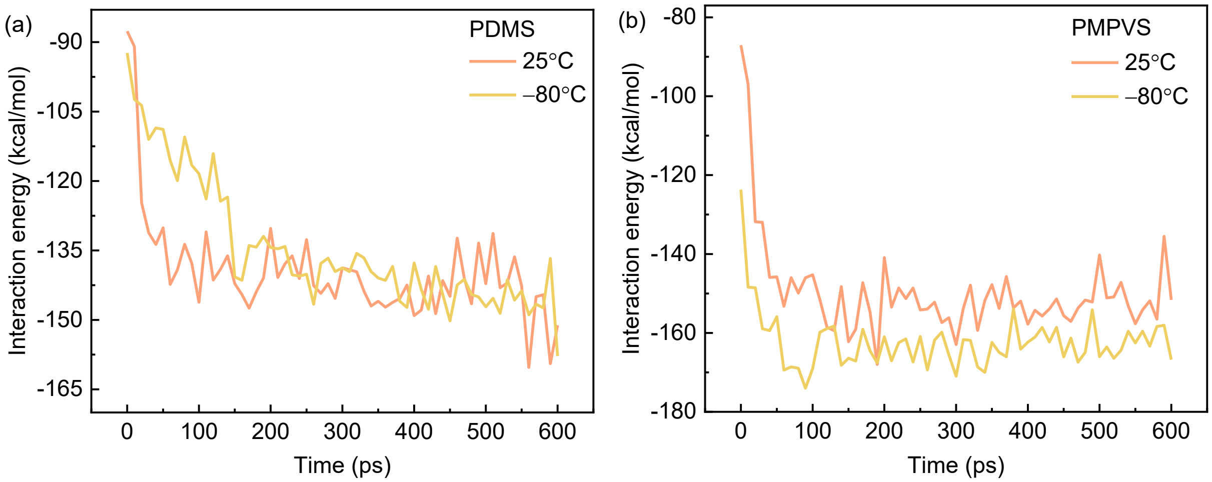


**Figure S16. The interaction energies between (a) PDMS and $SiO_2$ and (b) PMPVS and $SiO_2$ at 25°C and −80°C from 0 ps to 600 ps.**
